# Cu-chitosan nanoparticle boost defense responses and plant growth in maize (*Zea mays* L.)

**DOI:** 10.1038/s41598-017-08571-0

**Published:** 2017-08-29

**Authors:** Ram Chandra Choudhary, R. V. Kumaraswamy, Sarita Kumari, S. S. Sharma, Ajay Pal, Ramesh Raliya, Pratim Biswas, Vinod Saharan

**Affiliations:** 1 Department of Molecular Biology and Biotechnology, Rajasthan College of Agriculture, Maharana Pratap University of Agriculture and Technology, Udaipur, Rajasthan 313 001 India; 2 Department of Plant Pathology, Rajasthan College of Agriculture, Maharana Pratap University of Agriculture and Technology, Udaipur, Rajasthan 313 001 India; 3 Department of Chemistry and Biochemistry, College of Basic Sciences and Humanities, Chaudhary Charan Singh Haryana Agricultural University, Hisar, Haryana 125 004 India; 4 Department of Energy, Environmental and Chemical Engineering, Washington University in St. Louis, MO, 63130 USA

## Abstract

In agriculture, search for biopolymer derived materials are in high demand to replace the synthetic agrochemicals. In the present investigation, the efficacy of Cu-chitosan nanoparticles (NPs) to boost defense responses against Curvularia leaf spot (CLS) disease of maize and plant growth promotry activity were evaluated. Cu-chitosan NPs treated plants showed significant defense response through higher activities of antioxidant (superoxide dismutase and peroxidase) and defense enzymes (polyphenol oxidase and phenylalanine ammonia-lyase). Significant control of CLS disease of maize was recorded at 0.04 to 0.16% of Cu-chitosan NPs treatments in pot and 0.12 to 0.16% of NPs treatments in field condition. Further, NPs treatments exhibited growth promotry effect in terms of plant height, stem diameter, root length, root number and chlorophyll content in pot experiments. In field experiment, plant height, ear length, ear weight/plot, grain yield/plot and 100 grain weight were enhanced in NPs treatments. Disease control and enhancement of plant growth was further enlightened through Cu release profile of Cu-chitosan NPs. This is an important development in agriculture nanomaterial research where biodegradable Cu-chitosan NPs are better compatible with biological control as NPs “mimic” the natural elicitation of the plant defense and antioxidant system for disease protection and sustainable growth.

## Introduction

Environmental contamination has become a challenging issue because of uncontrolled and rampant use of synthetic agrochemicals for plant growth and protection^1^. The perpetual use of agrochemicals causes several adverse effects including, increased resistance in plant pathogenic microbes, negative impact on non-target organisms and deterioration of soil health^2, 3^. Globally, crops are severely affected by diseases which lead to qualitative and quantitative losses in agriculture^4^. Consequently, potential emphasis needs to be concentrated on development of biomaterial based biodegradable agrochemicals for effective and safe application in crops. Chitosan, a versatile biomaterial that is of a non-toxic, biocompatible and biodegradable nature, is being exploited in agriculture^5, 6^. It is well recognized as an antimicrobial^7, 8^, immuno modulatory^9–11^ and plant growth promotry agent^12, 13^. Higher physiological and biochemical responses of chitosan based NPs as compared to bulk chitosan is due to its high surface to volume ratio and surface charge^14–16^. Hence, chitosan based NPs have been used for various applications in agriculture including plant growth^13–18^. Recently, chitosan based NPs have been evaluated as potent inducer of antioxidant and defense enzymes^17, 19^. Transcript analysis of chitosan NPs treated plants depicted that increased level of defense responses was due to high expression of defense related genes. These findings supported the enhanced innate immunity of plants by chitosan component of NPs^18^. In our previous studies, we have reported Cu-chitosan NPs as an effective antifungal and plant growth promotry agent^15, 20^. Further studies revealed that application of Cu-chitosan NPs enhanced maize seedling growth by mobilizing reserve food through the enhanced activities of α﻿-amylase and protease^21^. To comprehend the dynamic bioactivities of Cu-chitosan NPs, which makes them more bioactive to other chitosan based NPs, we ought to understand the physicochemical properties of these NPs. Cu-chitosan NPs demonstrates porous network in which Cu is entrapped in the pores^15^. We strongly reckon that the porous architecture of chitosan NPs slowly releases Cu from the nanostructures. Therefore, we presupposed that after inflowing of Cu-chitosan NPs to plant cells, the contact of Cu to cellular system is long lasting^14, 15, 20^. As we acquainted, Cu is well established in plants as a key structural and catalytic component in various enzymes of electron transfer and redox reactions, thus, crucial for boosting plant growth^22, 23^. Therefore, sustained releases of Cu from NPs grave for accelerating various metabolic processes in plant growth during various development stages. Moreover, in acidic pH environment of target site, chitosan porous network dissolved and entrapped Cu release faster^14, 15^. Alongside, it has been envisaged that establishment of acidic pH during infection of plant pathogenic fungi, faster releases of Cu may wield strong fungicidal activity against fungal pathogens^14, 15, 24^. Thus Cu-chitosan NPs expressed a far elevated and diverge bioactivity as compared to sole chitosan based NPs. Up to now, rudimentary studies have been performed to induce the plant innate system for plant defense and subsequent higher growth and yield by NPs applications, thus, need further study of Cu-chitosan NPs for its effect on plant growth and protection for its comprehensive application in crop. World-wide, maize is an important food crop but is prone to various fungal diseases like curvularia leaf spot (CLS) disease caused by *Curvularia lunata*, which alone causes yield loss up to 60%^25^. Many strategies have been applied to control CLS disease using chemicals and other bio-agent but there is no report on evaluation of Cu-chitosan NPs against CLS disease in maize.

In the present investigation, we report for the first time the efficacy of Cu-chitosan NPs to induce the defense responses against CLS disease in maize, and to promote sustainable plant growth under net house and field conditions. Our results convincingly establish Cu-chitosan NPs as a potent inducer of systemic acquired resistance for effective control of CLS disease of maize and a plant growth promotry agent.

## Results

### Cu-chitosan NPs

The NPs used in present study had almost same characteristics of mean hydrodynamic diameter 361.3 ± 2.1 nm, zeta-potential +22.1 mV and 0.20 PDI value (Fig. S1) as reported previously in DLS study (Fig. S2)^15, 21^. DLS data verified the stability (sufficent repulsion between positively charged NPs) and monodispersed nature (low PDI) of NPs in aqueous. In our earlier study, these NPs were well charaterized for various physico-chemical properties like interaction of chitosan to TPP and Cu by fourier transform infrared spectroscopy (FT-IR) (Fig. S2), internal architecture by transmission electron microscopy (TEM) (Fig. S3), external architacture by scanning electron microscopy (SEM) (Fig. S3) and elemental analysis by energy dispersive X-ray spectroscopy (EDX) (Fig. S4)^15, 21^. Based on the physico-chemical charaterizations, a hypothatical model was proposed for understanding of structural and synthesis aspects of Cu-chitosan NPs (Fig. S5)^15^. Affinity of bulk chitosan towards metals, espacially Cu, has been well described in literature and strong evidences suggest that Cu-chitosan exist as metal-polymer complex through C-N bonding^26, 27^. Further, Cu-chitosan nano-complex has also been broadly studied through FT-IR, TEM, SEM and EDX. FT-IR spectrum of Cu–chitosan NPs strongly evident that peaks at 1636 cm^−1^ (CONH_2_) and 1550 cm^−1^ (NH_2_) were sharper and shifted to 1631 and 1536 cm^−1^ which denotes Cu bonding with chitosan nanostructers (Fig. S2)^15, 20^. TEM micrographs have visibly described Cu deposition into highly porous network of chitosan nanomaterials (Fig. S3)^15^. SEM-EDX analyses further inferred the higher deposition of Cu into porous surface of Cu–chitosan NPs (Fig. S4)^15^. Higher zeta-potential, low PDI value and embedded Cu endowed Cu-chitosan NPs a highly biologically active nanomaterial^14, 15^. These laboratory synthesized stable NPs were used in present study to evaluate their *in vitro* antifungal activity, effect on antioxidant and defense enzymes, disease control, plant growth and yield promotion in maize (Table 1).

**Table 1:** Experimental outline.

Experiment	Analysis/method	Remarks
Synthesis of Cu-chitosan NPs	Ionic gelation method^15^	Cu-chitosan NPs were synthesized
*In-vitro* Cu release	Using AAS^15^	Cu release from Cu-chitosan NPs was evaluated with respect to pH and time
Pot experiment		
Antioxidant and defense enzymes assay	Methods described by Giannopolitis and Ries^52^; Chance and Maehly^49^; Moerschbacher *et al*.,^51^; Taneja and Sachar^50^	Activities of SOD, POD, PAL and PPO were estimated
Chlorophyll content (a, b)	As described by Stangarlin *et al*.^47^	Chlorophyll content (a, b) was quantified
Disease assessment	Using 1 to 9 standard disease rating scale	DS and PEDC were calculated
Copper content in leaves	AAS method described by Adrian^48^.	Cu content in leaves was determined
Field experiment		
Days to 50%, tasseling, silking, ear leaf senescence and number of leaves/plant, plant height, ear length, ear weight/plot, grain yield/plot and 100 grain weight	Maize descriptor^53^	Crop yield assessment
Statistical analysis	JMP version-12 using Tukey–Kramer HSD test	Significant difference between treatment *(p* = 0.05) were calculated

### Cu release profile

**Figure 1:**
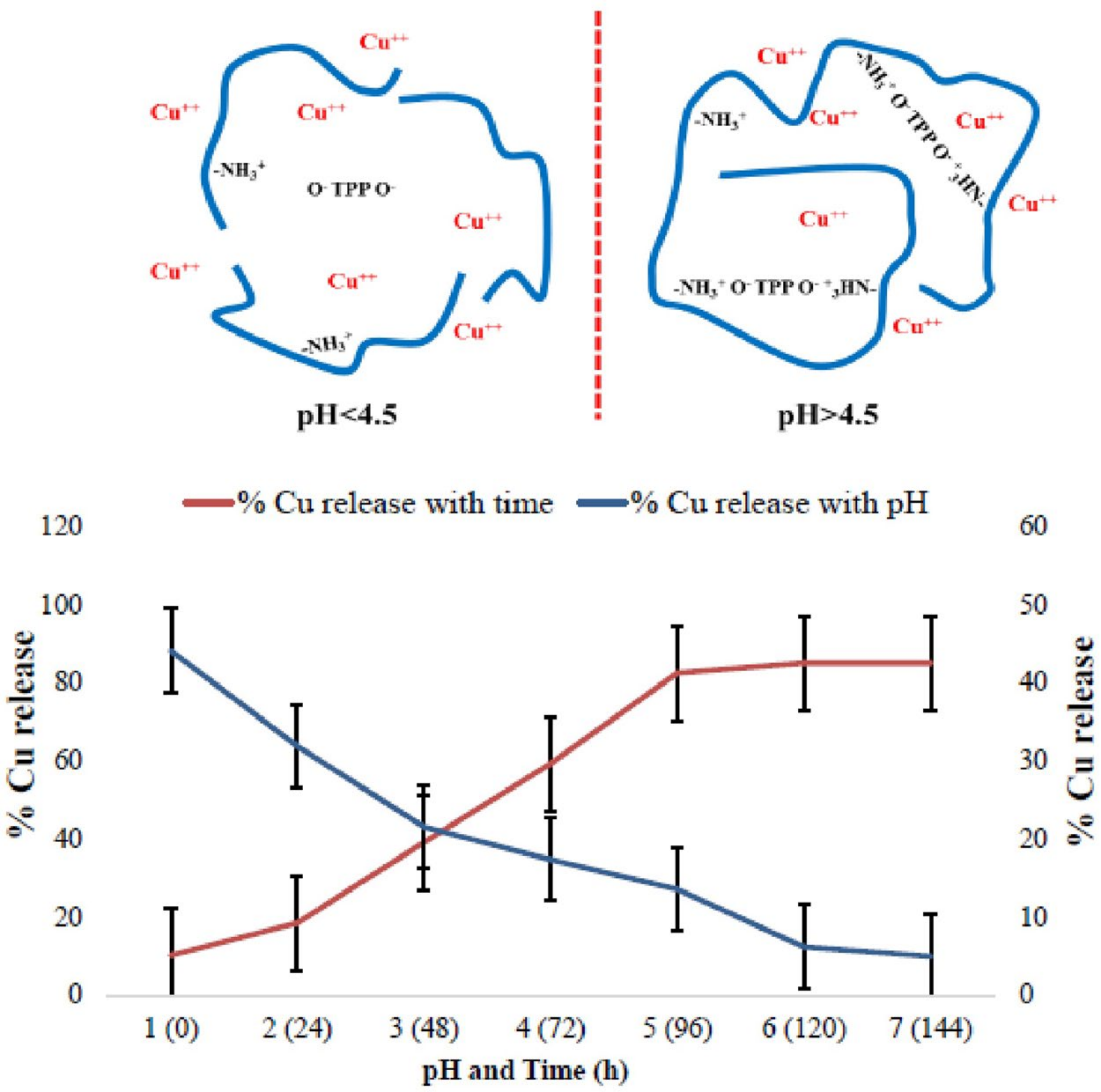
*In-vitro* Cu release from Cu-chitosan NPs at different pH and time. Each value is mean of triplicates and each replicate consisted of 3 samples.

Release of Cu from Cu-chitosan NPs was studied in the pH range 1 to 7 (Fig. 1). With decrease in pH from 3 to 1, release of Cu increased rapidly from 21.5 to 44.11% due to protonation of amino group of chitosan. At pH above 6, release of Cu drastically decreased (4.94%) due to deprotonation of amino group of chitosan (Fig. 1; Table S7). Release study was further continued at 4.5 pH with respect to time. Cu release increased slowly and steadily with time and at 96 h ~85% of Cu released from Cu-chitosan NPs (Fig. 1; Table S7). The release profile indicates that acidic pH expedited the Cu release and over time (at pH 4.5), a slow and sustained release of Cu from Cu-chitosan NPs was evident.

### *In vitro* antifungal activity of Cu-chitosan NPs

Cu-chitosan NPs comprehensively inhibited *in vitro* mycelial growth of *C. lunata*. Cu-chitosan NPs (0.12–0.16%) significantly inhibited mycelial growth from 50.0 to 52.7% as compared to all other treatments (Fig. S6 and Table S1).

### Effect of Cu-chitosan NPs on activities of antioxidant and defense enzymes in pot experiment

**Figure 2:**
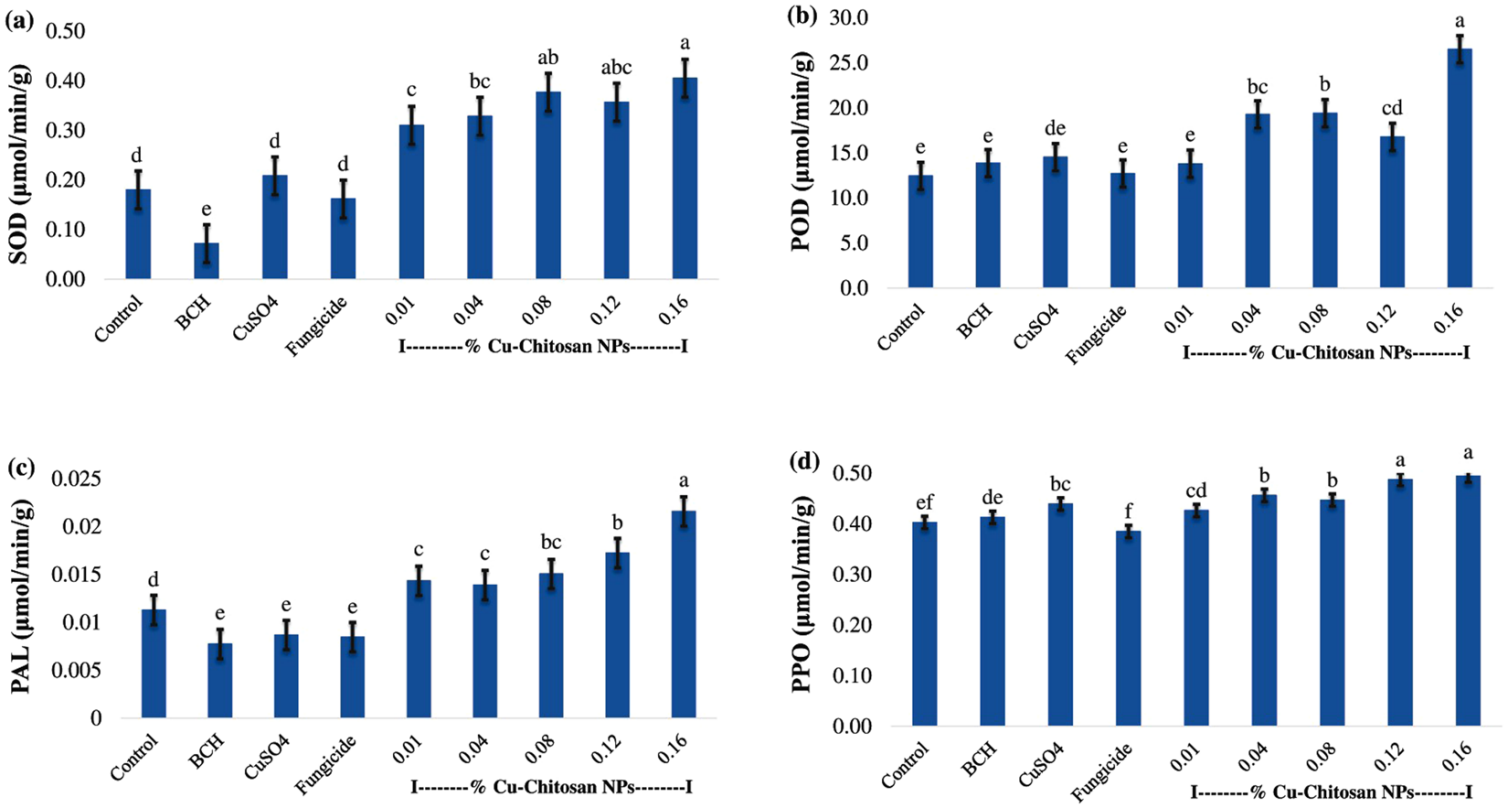
Effect of Cu-chitosan NPs on (**a**) SOD (**b**) POD (**c**) PAL (**d**) PPO enzymes activity in maize plant leaves after 24 h of foliar spray. Each value is mean of triplicates and each replicate consisted of 3 plants samples and same letter in the graph of each treatment is not significantly different at *p* = 0.05 as determined by Tukey–Kramer HSD, control with water. BCH (bulk chitosan, 0.01%) dissolved in 0.1% acetic acid. CuSO_4_ (0.01%) and fungicide (0.01% of Bavistin).

To estimate the activities of antioxidant and defense enzymes, leaf samples were collected after 24 h of foliar treatments. Application of NPs substantially induced the enzyme activities in leaves. SOD activity was significantly higher in all the treatments of NPs (Fig. 2a; Table S8). Similarly, 1.5–2 folds higher POD activity was recorded in 0.04 to 0.16% NPs treated plant leaves as compared to control and bulk chitosan treated plants (Fig. 2b; Table S8). Likewise, Cu-chitosan NPs treated plants leaves showed 2–3 folds increased PAL activity as compared to bulk chitosan treatment (Fig. 2c; Table S8). The activity of PPO was also enhanced by NPs treatments (0.12 and 0.16%) as compared to control (water), bulk chitosan and CuSO_4_ treatments (Fig. 2d; Table S8).

### Effect of Cu-chitosan NPs on CLS disease in pot experiment

**Figure 3:**
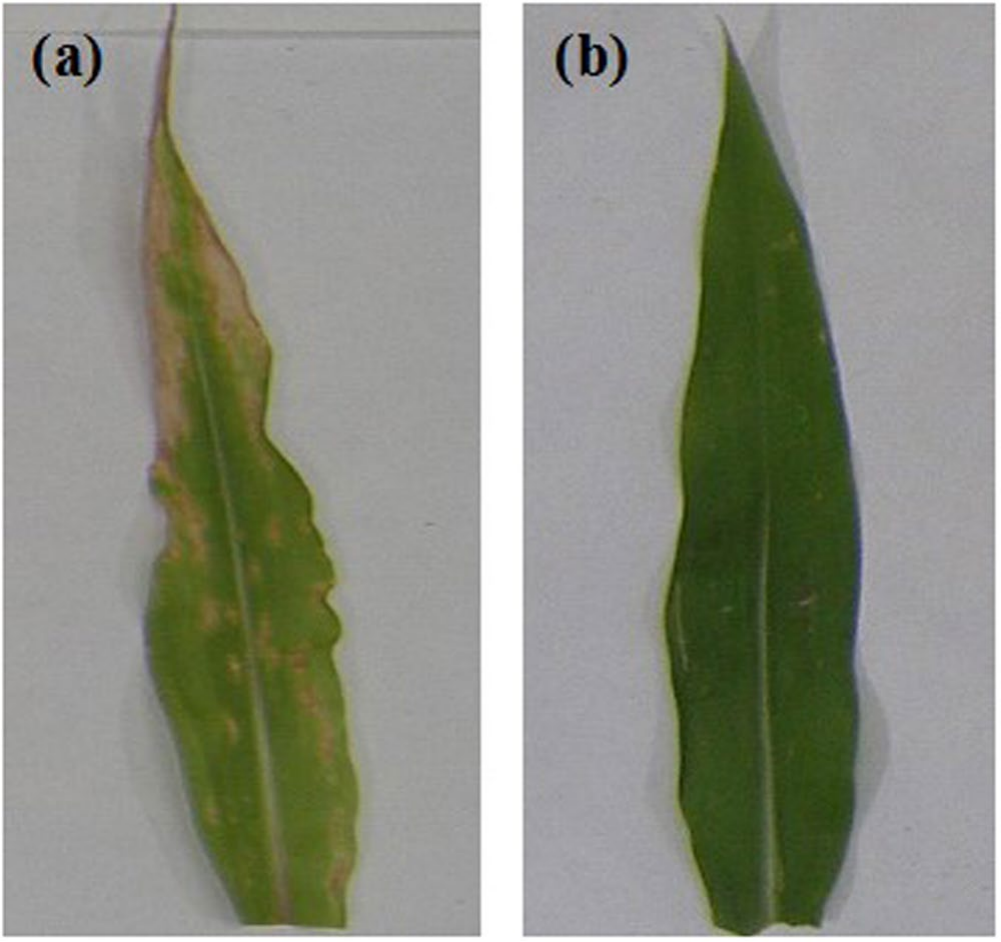
Symptoms of CLS disease on maize plant leaf in pot experiments (**a**) large necrotic lesion in control (**b**) micro lesions on Cu-chitosan NPs (0.16%) treated leaf.

In pot experiment, symptoms of CLS disease initiated after 3–4 days of fungal inoculation in control plants. The early appeared small chlorotic spot gradually extended into large eye shaped lesion, leading to the formation of leaf necrosis (Fig. 3a). Contrarily, in NPs treated plants, the disease symptoms in the form of small lesions without chlorosis were visualized after 7–8 days of fungal inoculation (Fig. 3b). The spread and severity of disease was also slow in proceeding days. After 15 days of inoculation, data for DS and PEDC were recorded. DS decreased with increasing concentrations of NPs as compared to other treatments (Table 2; Table S3). Commercially available fungicide (0.01% Bavistin), used as positive control, showed 29.3% DS. All the plants treated with 0.04 to 0.16% Cu-chitosan NPs showed significantly lower DS to an extant of 24.6–22.6%. The Cu-chitosan NPs at 0.04–0.16% significantly controlled CLS disease as depicted by higher value of PEDC (Table 2; Table S3).

**Table 2:** Effect of Cu-chitosan NPs on CLS disease in pot condition.

Treatment (%)	DS (%)^A^	PEDC (%)^A^
Control (water)	44.00 ± 1.15^a^	00.00 ± 0.00^e^
BCH (0.01)	32.67 ± 0.66^b^	25.72 ± 1.01^d^
CuSO_4_ (0.01)	32.00 ± 1.15^bc^	27.24 ± 2.31 ^cd^
Fungicide (0.01)	29.33 ± 0.66^bc^	33.31 ± 0.85^bc^
Cu-chitosan NPs		
0.01	28.67 ± 0.16^c^	34.83 ± 0.87^b^
0.04	24.67 ± 0.32^d^	43.86 ± 2.06^a^
0.08	24.67 ± 0.21^d^	43.93 ± 0.78^a^
0.12	23.33 ± 0.26^d^	46.97 ± 0.76^a^
0.16	22.67 ± 0.56^d^	48.48 ± 0.76^a^

Disease data were recorded after 15 days of inoculation using 1 to 9 standard disease rating scale. ^A^Each value is mean of triplicates and each replicate consisted of 3 plants samples. Mean ± SE followed by same letter is not significantly different at *p* = 0.05 as determined by Tukey–Kramer HSD. BCH (bulk chitosan) dissolved in 0.1% acetic acid and fungicide (0.01% of Bavistin).

### Effect of Cu-chitosan NPs on plant growth in pot experiment

**Figure 4:**
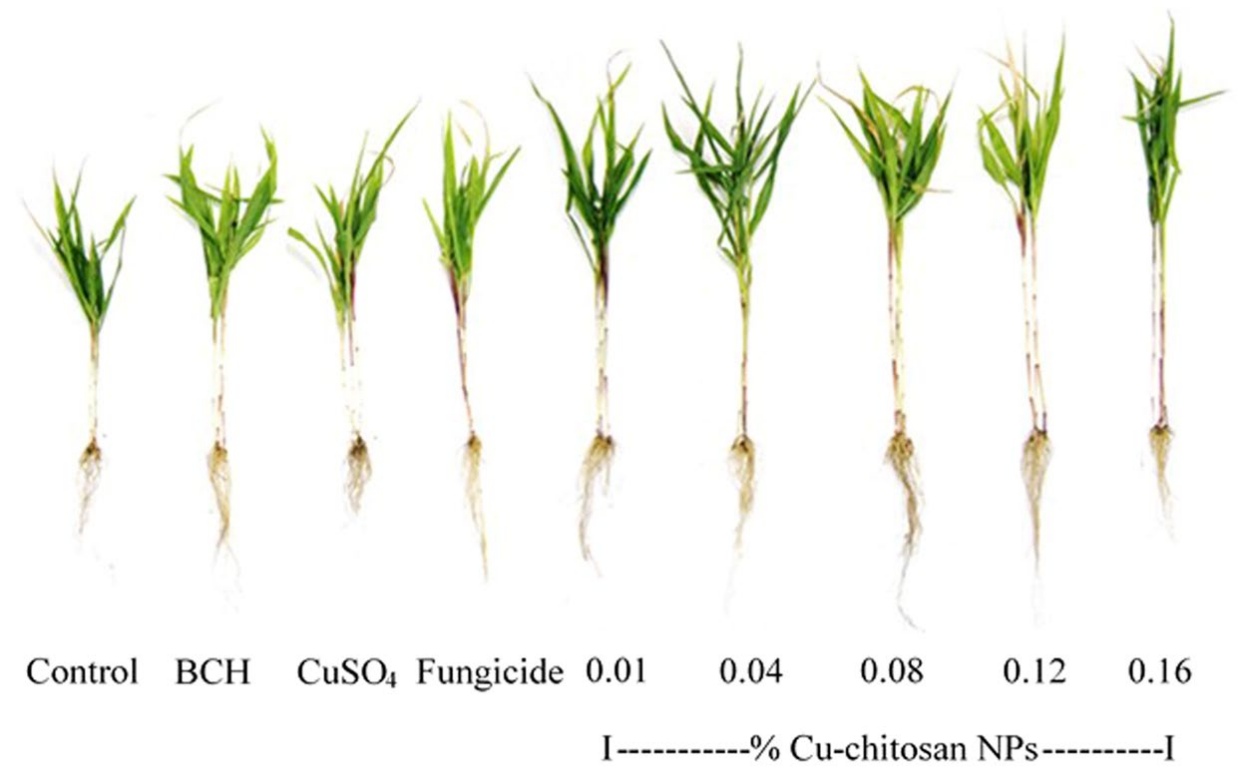
Effect of Cu-chitosan NPs on plant growth of maize in pot condition.

**Figure 5:**
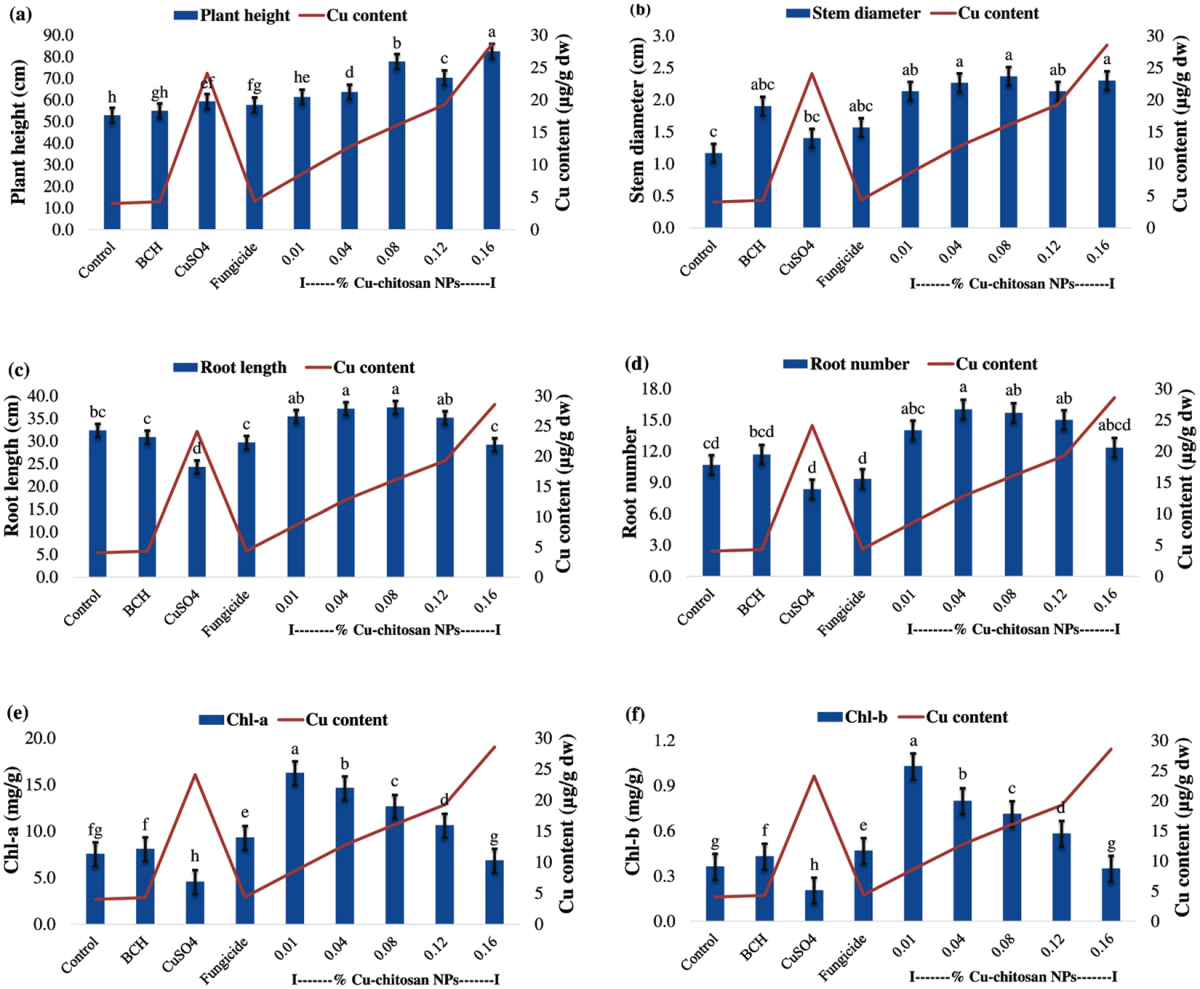
Effect of Cu-chitosan NPs on (**a**) plant height (**b**) stem diameter (**c**) root length (**d**) root number (**e**) chlorophyll-a and (**f**) chlorophyll-b content. Each value is mean of triplicates and each replicate consisted of 3 plants samples and same letter in the graph of each treatment is not significantly different at *p* = 0.05 as determined by Tukey–Kramer HSD, control with water. BCH (bulk chitosan, 0.01%) dissolved in 0.1% acetic acid. CuSO_4_ (0.01%) and fungicide (0.01% of Bavistin).

To evaluate the effect of NPs on plant growth, various growth characteristics namely plant height, stem diameter, root length, root number and chlorophyll content were recorded. Statistical analyses showed that Cu-chitosan NPs significantly enhanced the growth of maize plants in pot experiments as compared to control, bulk chitosan, CuSO_4_ and fungicide treatments (Fig. 4). Significantly higher values of plant height, stem diameter, root length and root number were recorded in 0.01 to 0.12% NPs treated plants (Fig. 5a–d; Table S9). A significant increase in chlorophyll a and b content (10.58 to 16.22 mg/g and 0.58 to 1.03 mg/g) was recorded in 0.01 to 0.12% of NPs treatments. In CuSO_4_ treatment, chlorophyll a and b content were found minimum (4.53 and 0.20 mg/g) followed by 0.16% NPs (6.81 and 0.35 mg/g) treatment (Fig. 5e,f; Table S9). To illustrate the possible association between plant growth and Cu, Cu content was estimated in treated plant leaves by AAS. Increasing concentrations of Cu-chitosan NPs (0.01–0.16%) showed increased Cu content (8.6–28.5 µg/g dry weight) in treated leaves (Table 3; Table S4). CuSO_4_ (0.01%) treated plant leaves had 24.1 µg/g dry weight of Cu, whereas in control (water) and bulk chitosan treatment, same content was observed (Table 3; Table S4).

**Table 3:** Cu content in maize leaves in various treatments.

Treatment (%)	Cu content^A^ (µg/g dw)
Control (water)	4.02 ± 0.37 ^g^
BCH (0.01)	4.28 ± 0.24 ^g^
CuSO_4_ (0.01)	24.10 ± 0.67^b^
Fungicide (0.01)	4.37 ± 0.37 ^g^
Cu-chitosan NPs	
0.01	8.60 ± 0.47 ^f^
0.04	12.77 ± 0.33^e^
0.08	16.07 ± 0.13^d^
0.12	19.25 ± 0.61^c^
0.16	28.55 ± 0.50^a^

Data were recorded in 3^rd^ leaf after harvest. ^A^Each value is mean of triplicates and each replicate consisted of 3 plants samples. Mean ± SE followed by same letter is not significantly different at *p* = 0.05 as determined by Tukey–Kramer HSD. BCH (bulk chitosan, 0.01%) dissolved in 0.1% acetic acid and fungicide (0.01% of Bavistin).

### Effect of Cu-chitosan NPs on CLS disease and crop yield

Data for DS and PEDC were recorded after 20 days of inoculation. At 0.12 and 0.16% of NPs treatments, plant showed comparatively less disease severity to other concentration of NPs, control (water), bulk chitosan, CuSO_4_ and fungicide (Table 4; Table S5). Likewise, disease control was also considerably higher at higher concentration of NPs i.e. 0.12 and 0.16% (Table 4; Table S5). In field experiment, days to 50% tasseling, silking ear leaf senescence and number of leaves per plant were not affected by NPs treatments and remain same as with other treatments. Plant height and ear length was marginally higher in NPs treatments, whereas, ear weight/plot, grain yield/plot and 100 grain weight were significantly higher in 0.12 to 0.16% of NPs treatments (Table 5; Table S6).

**Table 4:** Effect of Cu-chitosan NPs on CLS disease in field condition.

Treatment (%)	DS (%)^A^	PEDC (%)^A^
Control (water)	64.26 ± 0.94^a^	00.00 ± 0.00^d^
BCH (0.01)	48.18 ± 0.43^b^	25.00 ± 0.81^c^
CuSO_4_ (0.01)	47.53 ± 0.61^bc^	26.00 ± 1.66^bc^
Fungicide (0.01)	46.99 ± 0.23^bc^	26.82 ± 1.43^bc^
Cu-chitosan NPs		
0.01	46.42 ± 0.31^bc^	27.72 ± 0.98^bc^
0.04	46.26 ± 0.16^bc^	27.96 ± 1.22^bc^
0.08	45.90 ± 0.45^bc^	28.53 ± 0.09^bc^
0.12	44.71 ± 0.80^cd^	30.42 ± 0.28^ab^
0.16	42.48 ± 1.08^d^	33.88 ± 1.10^a^

Disease data were recorded after visible appearance of symptoms following 20 days of inoculation using 1 to 9 standard disease rating scale. ^A^Each value is mean of triplicates and each replicate consisted of 10 plants samples. Mean ± SE followed by same letter is not significantly different at *p* = 0.05 as determined by Tukey–Kramer HSD. BCH (bulk chitosan) dissolved in 0.1% acetic acid and fungicide (0.01% of Bavistin).

**Table 5:** Effect of Cu-chitosan NPs on growth parameter of maize in field condition.

Treatment (%)	Days to 50% tasseling	Days to 50% silking	Days to 50% ear leaf senescence	Number of leaves per plant^A^	Plant height (cm)^A^	Ear length (cm)^A^	Ear weight (kg/plot)^B^	Grain yield (kg/plot)^B^	100 grain weight (g)^B^
Control (water)	53.66 ± 0.33^a^	55.66 ± 0.33^a^	78.33 ± 0.88^bc^	12.66 ± 0.38^bc^	176.33 ± 1.83^c^	19.66 ± 0.88^b^	2.83 ± 0.09^ab^	2.08 ± 0.03^abcd^	25.55 ± 0.29^b^
BCH (0.01)	54.33 ± 0.33^a^	58.66 ± 0.33^a^	77.67 ± 0.33^c^	12.22 ± 0.96^bc^	186.22 ± 1.3^b^	21.00 ± 1.15^ab^	2.69 ± 0.06^abc^	2.26 ± 0.15^abcd^	25.68 ± 0.30^b^
CuSO_4_ (0.01)	54.33 ± 0.88^a^	57.66 ± 0.33^a^	80.67 ± 0.33^abc^	11.33 ± 0.38^c^	185.77 ± 2.43^b^	22.66 ± 0.88^ab^	1.89 ± 0.12^d^	1.46 ± 0.18^d^	25.09 ± 0.69^b^
Fungicide (0.01)	54.00 ± 0.57^a^	56.33 ± 0.88^a^	79.33 ± 0.33^abc^	11.22 ± 0.11^c^	189.88 ± 2.99^ab^	22.00 ± 1.52^ab^	2.10 ± 0.09 ^cd^	1.58 ± 0.06 ^cd^	26.70 ± 0.41^b^
Cu-chitosan NPs									
0.01	53.33 ± 0.33^a^	57.00 ± 1.00^a^	81.33 ± 0.66^abc^	13.44 ± 0.22^bc^	190.22 ± 1.82^ab^	22.66 ± 0.88^ab^	2.42 ± 0.12^bcd^	1.87 ± 0.09^abcd^	26.82 ± 0.37^b^
0.04	53.33 ± 0.33^a^	56.66 ± 0.66^a^	82.00 ± 1.00^ab^	13.77 ± 0.40^ab^	194.00 ± 0.19^ab^	22.66 ± 1.76^ab^	2.37 ± 0.21^bcd^	1.79 ± 0.30^bcd^	25.77 ± 0.35^b^
0.08	54.00 ± 0.57^a^	57.00 ± 0.57^a^	82.67 ± 1.33^a^	15.88 ± 0.55^a^	198.11 ± 2.45^a^	26.33 ± 0.88^a^	2.70 ± 0.03^abc^	2.38 ± 0.15^abc^	26.91 ± 0.53^b^
0.12	53.66 ± 0.66^a^	56.33 ± 0.88^a^	80.67 ± 0.33^abc^	12.66 ± 0.33^bc^	198.00 ± 1.30^a^	24.00 ± 1.00^ab^	3.09 ± 0.21^a^	2.61 ± 0.21^ab^	29.23 ± 0.46^a^
0.16	53.66 ± 0.66^a^	57.00 ± 0.57^a^	79.67 ± 0.88^abc^	12.00 ± 0.38^bc^	191.00 ± 1.07^ab^	25.00 ± 1.00^ab^	3.10 ± 0.03^a^	2.69 ± 0.12^a^	29.80 ± 0.59^a^

Various growth parameters were recorded at 80 days^A^ and 95 days^B^of crop. Each value is mean of triplicates and each replicate consisted of 10 plants samples. Mean ± SE followed by same letter is not significantly different at *p* = 0.05 as determined by Tukey–Kramer HSD. BCH (bulk chitosan) dissolved in 0.1% acetic acid and fungicide (0.01% of Bavistin).

## Discussion

Among foliar diseases, CLS is common in maize growing countries and first reported from North Carolina and Georgia^28^. Disease prevalent in hot, humid areas and the severity of the disease depends on environmental conditions and susceptibility of maize lines. Infected leaves develop white circular spots with dark/ brown marginal rings, that often coalesce to form larger infected areas, subsequently affected yellow leaves dried, cause significant damage up to 60%^29^. Chitosan NPs have previously been reported as immune modulator through induction of antioxidant/defense enzymes activity in tea and finger millet plants^11, 19^. Before conducting the pot and field experiments for defense responses and plant growth, *in vitro* antifungal test was conducted to depict the degree of growth inhibition of Cu-chitosan NPs against *C. lunata*. A considerable % inhibition of mycelial growth was noticed against *C. lunata* in the experiment (Table S1). In our previous study, same batch of Cu-chitosan NPs were found effective in inhibiting mycelial growth of *Alternaria solani* and *Fusarium oxysporum* in *in vitro* experiments^15^. In present study, foliar application of Cu-chitosan NPs in pot experiments substantially induced antioxidant/defense enzyme activity in maize leaves. NPs treated plant leaves showed 4–6 fold higher activity of SOD as compared to bulk chitosan (Fig. 2a). The higher activity of SOD effectively converts highly toxic superoxide radicles into less toxic H_2_O_2_ species^30^. A significantly higher activity of POD, a key enzyme to scavenge H_2_O_2_ into H_2_O and O_2_, was also recorded in NPs treated leaves (Fig. 2b). The elevated activities of SOD and POD after NPs treatments might be responsible for balancing, degeneration, and scavenging of reactive oxygen species (ROS) to protect plant from oxidative stress during pathogen invasion.

In Cu-chitosan NPs treated plant leaves, PAL activity also persuaded from 46.15 to 66.66% and PPO activity increased from 3.05 to 16.39% as compared to bulk chitosan treatment (Fig. 2c,d). The increased activity of POD, PAL and PPO might be associated with production of suberin, melanin and lignin^31, 32^ for cell wall strengthening which further acts as a mechanical barrier to invading plant pathogen^31–34^.

To test the efficacy of Cu-chitosan NPs against CLS disease, pot and field experiments were conducted by inoculating highly sporulating inoculum of *C. luanta*. Sorghum grain medium was used to grow the fungal culture to achieve higher mycelial growth and sporulation and to maintain the desire pathogenicity of the fungal inoculum. The detailed methodology has been explained elsewhere^35^. This method is widely adopted to develop CLS disease artificially in pot and field conditions. As already mentioned, sorghum grain medium gave excellent results to achieve adequate mycelial growth and sporulation as compared to other grain like wheat, barley, rice, maize etc. The content and type of sorghum carbon source may be expected more suitable for rapid mycelial growth and sporulation as compared to maize grain. In pot experiment, DS and PEDC were recorded to determine the efficacy of Cu-chitosan NPs against CLS disease. A significant control of CLS disease was recorded on Cu-chitosan NPs treatments (0.04–0.16%) as compared to others (Table 2). These Cu-chitosan NPs (0.08–0.12%) have previously been reported very effective against early blight and *Fusarium* wilt of tomato^15^. The defense response of Cu-chitosan NPs might be due to direct activity like (a) through membrane destruction by electrostatic interaction of chitosan with microbial cell surface^6^ (b) positively charged NPs could binds to DNA/RNA which affects transcription and translation process and inhibit fungal proliferation^6^. On another side, indirect activity might be exerted by chitosan through aroused plant immune response by enhanced activities of antioxidant and defense enzymes^36^. Furthermore, we foresee that Cu-chitosan NPs releases Cu rapidly in acidic pH (Fig. 1) which is created upon fungal infection and the released Cu may act weighty on the fungus^15^. Altogether Cu-chitosan NPs lead to abate *C. lunata* spreading and contributed resistance in maize plants against CLS disease through synergistic effect of chitosan and Cu^15, 24^.

**Figure 6:**
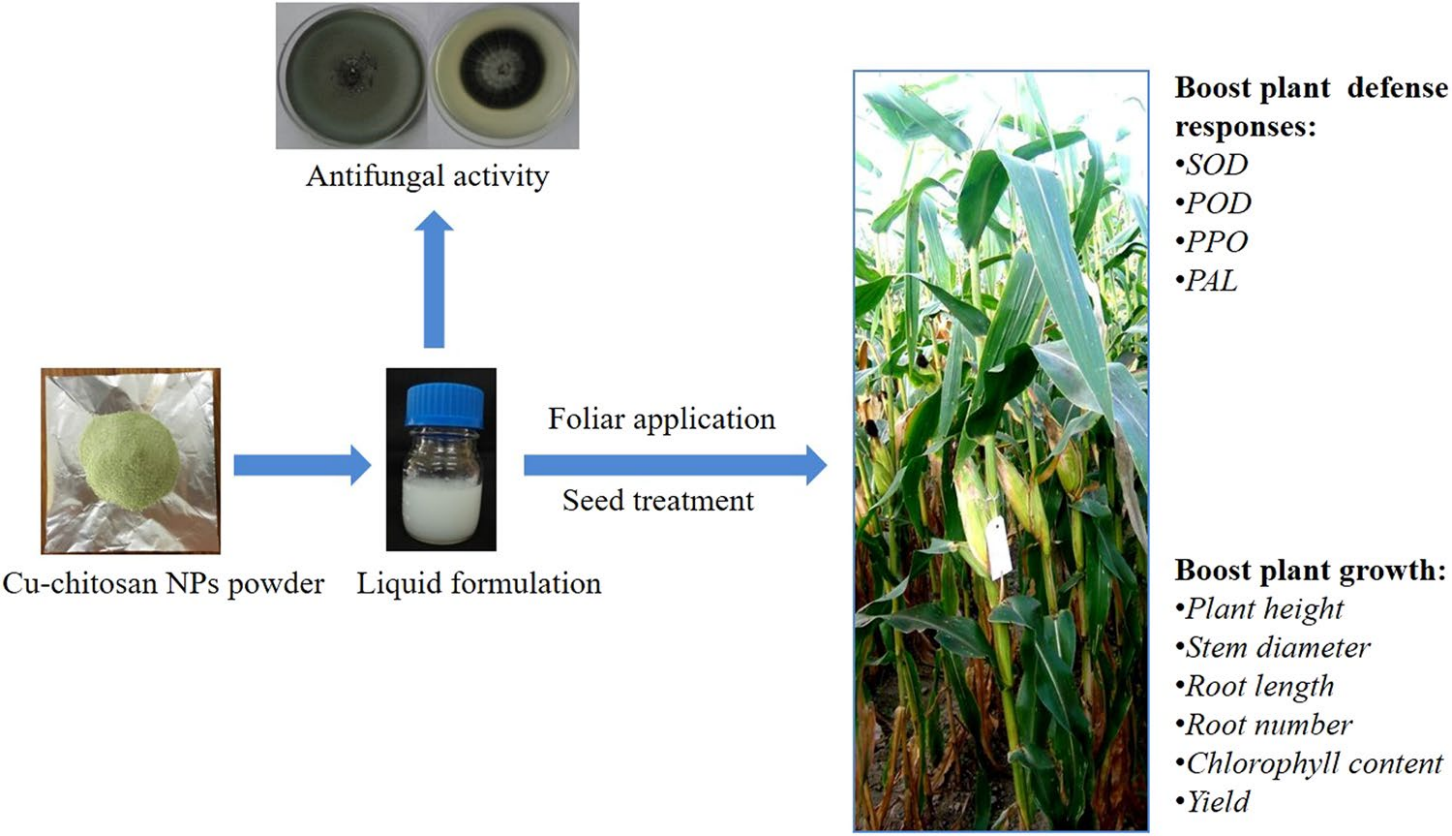
Application model of Cu-chitosan NPs induced defense responses and plant growth in maize.

These NPs significantly enhanced seedling growth of tomato^15^ and maize by mobilizing reserved food through higher activities of α-amylase and protease^21^. To take the advantage of growth promotry effect of Cu-chitosan NPs (as reported in our previous study), maize seeds were treated with Cu-chitosan NPs followed by foliar spray in pot experiment. Statistical analyses showed that Cu-chitosan NPs notably increased plant height, stem diameter, root length, root number and chlorophyll content (Fig. 5). However, at higher concentrations of Cu-chitosan NPs (0.16%) and CuSO_4_ (0.01%) treatment, chlorophyll content significantly decreased (Fig. 5e,f). It has previously been proposed that accumulation of Cu interferes with chlorophyll biosynthesis and cause deficiency of Mg and Fe^37–39^. Concomitantly, root length and root number was also affected at higher concentrations of NPs (0.16%) and CuSO_4_ (0.01%) (Fig. 5c,d). In AAS analyses, we quantified Cu content in treated plant leaves and allied it with plant growth characters (Table 3; Fig. 5). We disentangled that the trend of plant growth was virtually related with Cu content, and this is in line with our previous study^21^. The toxicity envisaged only on chlorophyll content, root length and root number at CuSO_4_ (0.01%) and 0.16% NPs treatments could be endowed by elevated accumulation of Cu (28.5 and 24.1 µg/g, Table 3). The accumulated level of Cu in present study is more than toxic level of Cu in maize leaves which is reported to be 20 µg/g^40^. Therefore, we expect that for conceivable plant growth, Cu uptake must be controlled to avoid its sudden exposure to plant cells^21^ which can be achieved by slow release of Cu from Cu-chitosan NPs (Fig. 1). In field experiment, disease control and yield was significantly influenced by NPs treatments. However in field, higher concentration of NPs (0.12 and 0.16%) showed significant disease control and galvanize grain yield. A sturdy conclusion could be towed from pot and field experiments that, application of Cu-chitosan NPs unquestionably control disease and boost plant growth and yield. Review of literature emphasized that during grain filling, an adequate level of antioxidant environment may contribute the mobilization of reserve food from leaf, stem etc to developing grain for uninterrupted grain filling^41, 42^. Studies have proved the antifungal activity of chitosan based NPs and its role in induction of defense enzymes^14^, however, the enhancement of growth and yield is not well understood. It is imperative to study further into reserve food mobilization and its interaction with NPs to optimize the application module of Cu-chitosan NPs for higher growth and yield of crop. Results in present study categorically claim that Cu-chitosan NPs act as antifungal agent, enhance the activities of antioxidant and defense enzymes which cohorts with plant growth and disease resistance in treated maize plants (Fig. 6).

## Conclusion

Demand of food/feed crop free from synthetic components has exponentially increased in recent years to avert hazardous effects of synthetic additives and to evade the development of resistance in pathogens. A new approach is imperative to be adapted to strengthen plant innate immunity to cope with mutating plant pathogens, reduce chemical use and alongside sustained plant growth. Cu-chitosan NPs have been proven as a promising plant protection and growth promotry agent in our past and recent studies. Its unique ability to sustain the plant growth under disease conditions makes it a very effective and usable agent. These bio-based nanomaterials could be pivotal towards sustainable agriculture without harming ecosystem. The synthesized NPs have immense potential to be commercially explored for agriculture use.

## Methods

The experiments performed in the study are summarized in Table 1 and details are in following headings.

### Materials

Chitosan (Mol. Wt. 50,000–190,000 and 80% N-deacetylation) and sodium tri-polyphosphate (TPP) were procured from Sigma-Aldrich, St. Louis, MO, USA. Chemicals for enzyme assay and other experiments were procured from HiMedia and SRL, Mumbai, India. The seeds of cultivar ‘*Surya local*’ were obtained from the Department of Plant Breeding and Genetics, Rajasthan College of Agriculture, Maharana Pratap University of Agriculture and Technology, Udaipur, India. Inoculum of *Curvularia lunata* was received from Department of Plant Pathology, Rajasthan College of Agriculture, Maharana Pratap University of Agriculture and Technology, Udaipur, India.

### Cu-Chitosan NPs

Cu-chitosan NPs were obtained as dry powder from the batch synthesized and used in our earlier study^15^. The obtained NPs were characterized for its mean hydrodynamic diameter, zeta-potential and polydispersity index (PDI) by dynamic light scattering (DLS) on Zetasizer ZS 90 (Malvern, UK) at 25 °C at a scattering angle of 90 ° in triplicate.

### *In-vitro* Cu release profile

*In-vitro* experiments were conducted to study the effect of pH and time on the release of Cu from Cu-chitosan NPs. In brief, freeze dried Cu-chitosan NPs were dispersed in deionized water with pH adjusted in the range of 1 to 7. The contents were centrifuged at 10,000 × g for 10 min and supernatants were collected for further analysis. Similarly, in separate experiments, the NPs were dispersed at 4.5 pH for 0, 24, 48, 72, 96, 120 and 144 h followed by centrifugation at 10,000 × g for 10 min. The supernatants, thus obtained, from both the experiments were analyzed for Cu contents using atomic absorption spectrophotometer (AAS 4141 model, Electronics Corp. of India Ltd., India).

### *In vitro* antifungal activity of Cu-chitosan NPs

Antifungal activity was evaluated by poisoned food technique^20, 43^ using Cu-chitosan NPs (0.01, 0.04, 0.08, 0.12 and 0.16%, w/v), control, bulk chitosan (0.01%,w/v), CuSO_4_ (0.01%,w/v) and commercially available fungicide (0.01% Bavistin, w/v). The treated plates were compared with the control to calculate the per cent inhibition of mycelia growth by using the formula given by Vincent^44^.

$$\begin{array}{c}{\rm{ \% }}\,{\rm{I}}{\rm{n}}{\rm{h}}{\rm{i}}{\rm{b}}{\rm{i}}{\rm{t}}{\rm{i}}{\rm{o}}{\rm{n}}=\text{Mycelial}\,{\rm{g}}{\rm{r}}{\rm{o}}{\rm{w}}{\rm{t}}{\rm{h}}\,{\rm{i}}{\rm{n}}\,{\rm{c}}{\rm{o}}{\rm{n}}{\rm{t}}{\rm{r}}{\rm{o}}{\rm{l}}\\ \qquad \qquad \,\,\,\qquad -\,{\rm{m}}{\rm{y}}{\rm{c}}{\rm{e}}{\rm{l}}{\rm{i}}{\rm{a}}{\rm{l}}\,{\rm{g}}{\rm{r}}{\rm{o}}{\rm{w}}{\rm{t}}{\rm{h}}\,{\rm{i}}{\rm{n}}\,\text{treatment}\times 100/\text{mycelial}\,{\rm{g}}{\rm{r}}{\rm{o}}{\rm{w}}{\rm{t}}{\rm{h}}\,{\rm{i}}{\rm{n}}\,{\rm{c}}{\rm{o}}{\rm{n}}{\rm{t}}{\rm{r}}{\rm{o}}{\rm{l}}\end{array}$$

### Pot experiment for disease assessment and plant growth

Seeds of disease susceptible maize cultivar ‘*Surya local*’ were surface sterilized with 10% sodium hypochlorite for 10 min and further treated for 4 h with different concentrations of Cu-chitosan NPs (0.01, 0.04, 0.08, 0.12 and 0.16%, w/v), control (water), bulk chitosan (0.01%,w/v), CuSO_4_ (0.01%,w/v) and commercially available fungicide (0.01% Bavistin, w/v). The treated seeds were dried and sown in earthen pots filled with standard clay type soil in net house condition by following standard agronomic practices. The plants were subjected to foliar spray of same treatments as did with seeds until runoff at 35 days of sowing. After 10 days of foliar treatments, inoculum of *C. lunata*, prepared on sorghum seed medium^35^, was inoculated on plants. In brief, *C. lunata* culture was seeded on medium papered using sorghum seeds and kept at 27 ± 1 °C for 15 days to achieve adequate mycelial growth and sporulation. Colonized grains were rinsed with water and conidial suspension was filtered through cheese cloth and diluted with water to a concentration of 5 × 10^3^ conidia ml^−1^. The inoculum was sprayed on plants by backpack sprayers for disease development in maize plants. Disease assessment was performed after appearance of symptoms on leaves at 15 days of inoculation. Disease Severity (DS) was recorded on 1 to 9 standard disease rating scale. Further, the DS and per cent efficacy of disease control (PEDC) were calculated by using the formula given by Chester^45^ and Wheeler^46^.

$${\rm{D}}{\rm{S}}=\text{Sum}\,{\rm{o}}{\rm{f}}\,{\rm{a}}{\rm{l}}{\rm{l}}\,{\rm{i}}{\rm{n}}{\rm{d}}{\rm{i}}{\rm{v}}{\rm{i}}{\rm{d}}{\rm{u}}{\rm{a}}{\rm{l}}\,{\rm{d}}{\rm{i}}{\rm{s}}{\rm{e}}{\rm{a}}{\rm{s}}{\rm{e}}\,{\rm{r}}{\rm{a}}{\rm{t}}{\rm{i}}{\rm{n}}{\rm{g}}\times 100/\text{total}\,{\rm{n}}{\rm{u}}{\rm{m}}{\rm{b}}{\rm{e}}{\rm{r}}\,{\rm{o}}{\rm{f}}\,{\rm{p}}{\rm{l}}{\rm{a}}{\rm{n}}{\rm{t}}\,{\rm{a}}{\rm{s}}{\rm{s}}{\rm{e}}{\rm{s}}{\rm{s}}{\rm{e}}{\rm{d}}\times {\rm{m}}{\rm{a}}{\rm{x}}{\rm{i}}{\rm{m}}{\rm{u}}{\rm{m}}\,{\rm{r}}{\rm{a}}{\rm{t}}{\rm{i}}{\rm{n}}{\rm{g}}.$$

$$\begin{array}{c}{\rm{PEDC}}=\text{Disease}\,\text{severity}\,{\rm{in}}\,{\rm{control}}-{\rm{disease}}\,{\rm{severity}}\,{\rm{in}}\,{\rm{treatment}}\,\times 100/\text{disease}\,{\rm{severity}}\,{\rm{in}}\,{\rm{control}}.\end{array}$$

The plants were harvested at maturity (95 days) to determine plant height, stem diameter, root length and root number. Chlorophyll a and b were quantified in 3^rd^ leaf after 24 h of foliar spray^47^. Cu content was also measured in 3^rd^ leaf of treated plant after harvest using AAS as described earlier^48^.

### Measurement of enzyme activity

Activity of antioxidant [superoxide dismutase (SOD) and peroxidase (POD)] and defense enzymes [phenylalanine ammonia-lyase (PAL) and polyphenol oxidase (PPO)] were estimated in 3^rd^ leaf after 24 h of foliar spray of various treatments. For enzymes extraction, 0.2 g samples were homogenized in 5 ml of extraction buffer (phosphate buffer for SOD and PPO at pH 7.4 and 6.8, respectively; tris-HCl buffer at pH 7.5 for POD and borate buffer at pH 8.8 for PAL). The homogenates were centrifuged at 10,000 × g for 20 min at 4 °C and supernatants were taken for enzymes assay. SOD (EC 1.15.1.1) activity was determined at 560 nm, as reduction of nitro-blue tetrazolium (NBT) as an indicator of superoxide anion production^39^. POD (EC 1.11.1.7) activity was measured spectrophotometrically as described by Chance and Maehly^49^ by oxidation of guaiacol in the presence of hydrogen peroxide. Increase in absorbance at 470 nm was recorded due to formation of tetra guaiacol. PPO (EC 1.10.3.1) was assayed according to Taneja and Sachar^50^ and activity was expressed as change in absorbance at 490 nm. PAL (EC 4.3.1.5) was estimated as described by Moerschbacher *et al*.^51^ where the deamination of L-phenylalanine to trans-cinnamic acid and ammonia was measured at 290 nm. Activities of all the enzymes were expressed in µmol/min/g tissue.

### Field experiment for disease assessment and crop yield

The field experiment was conducted in year 2016 (July to December) at research farm of Rajasthan College of Agriculture, Maharana Pratap University of Agriculture and Technology, Udaipur, India, (24.58° latitude, 73.70° longitude) on a standard clay type soil in randomized block design (RBD) with three replication. In each replication, seeds were grown in a plot (size 3.6 m^2^, where row to row and plant to plant space was 0.6 m and 0.2 m) having three rows (each 3 m). Seed treatment, foliar application, artificial inoculation, assessments of DS and PEDC were performed as explained in pot experiments. The plants were maintained as per standard agronomic practices. Observation of days to 50% tasseling, silking, ear leaf senescence and number of leaves/plant, plant height, ear length (at 80 days), ear weight/plot, grain yield/plot and 100 grain weight were recorded using maize descriptor.

### Statistical analysis

Statistical analysis of the data was performed with JMP software version 12. The significant differences among treatment groups were determined using the Turkey Kramer HSD at *p* = 0.05. All experiments were performed in three replications (triplicates) and each replication consisted of minimum three (for pot experiments) and ten samples (for field experiments) from randomly selected plants.

## Electronic supplementary material

Supplementary Information

## References

[1] Tilman D, Cassman KG, Matson PA, Naylor R, Polasky S (2002). Agricultural Sustainability and Intensive Production Practices. Nature.

[2] Kashyap PL, Xiang X, Heiden P (2015). Chitosan nanoparticle based delivery systems for sustainable agriculture. Int. J. Biol. Macromol..

[3] Zhan J, Thrall PH, Papaïx J, Xie L, Burdon JJ (2015). Playing on a pathogen’s weakness: using evolution to guide sustainable plant disease control strategies. Ann. Rev. Phytopathol..

[4] Savary, S., Ficke, A., Aubertot, J.-N. & Hollier, C. Crop losses due to diseases and their implications for global food production losses and food security. *Food Sec*. 1–19 (2012).

[5] Katiyar D, Hemantaranjan A, Singh B (2015). Chitosan as a promising natural compound to enhance potential physiological responses in plant: a review. Indian J. Plant Physiol..

[6] Xing K, Zhu X, Peng X, Qin S (2015). Chitosan antimicrobial and eliciting properties for pest control in agriculture: a review. Agronomy Sustain. Develop..

[7] Goy RC, Morais ST, Assis OB (2016). Evaluation of the antimicrobial activity of chitosan and its quaternized derivative on E. coli and S. aureus growth. Revista Brasileira de Farmacognosia.

[8] Kong M, Chen XG, Xing K, Park HJ (2010). Antimicrobial properties of chitosan and mode of action: a state of the art review. Int. J. Food Microbiol..

[9] Amborabé B-E, Bonmort J, Fleurat-Lessard P, Roblin G (2008). Early events induced by chitosan on plant cells. J. Exper. Bot..

[10] Popova E, Domnina N, Kovalenko N, Sokornova S, Tyuterev S (2016). Effect of chitosan and vanillin-modified chitosan on wheat resistance to spot blotch. Appl. Biochemi. Microbiol..

[11] Sathiyabama M, Manikandan A (2016). Chitosan nanoparticle induced defense responses in fingermillet plants against blast disease caused by *Pyricularia grisea* (Cke.) Sacc. Carbo. Poly..

[12] Kananont N, Pichyangkura R, Chanprame S, Chadchawan S, Limpanavech P (2010). Chitosan specificity for the *in vitro* seed germination of two Dendrobium orchids (Asparagales: Orchidaceae). Scientia Horticul..

[13] Sathiyabama M, Parthasarathy R (2016). Biological preparation of chitosan nanoparticles and its *in vitro* antifungal efficacy against some phytopathogenic fungi. Carbo. Polymers.

[14] Saharan, V. & Pal, A. Chitosan Based Nanomaterials in Plant Growth and Protection. *Springer Briefs in Plant Sci* (2016).

[15] Saharan V (2015). Synthesis and *in vitro* antifungal efficacy of Cu–chitosan nanoparticles against pathogenic fungi of tomato. Int. J. Biol. Macromol..

[16] Van SN, Minh HD, Anh DN (2013). Study on chitosan nanoparticles on biophysical characteristics and growth of Robusta coffee in green house. Biocat. Agric. Biotechnol..

[17] Sathiyabama M, Bernstein N, Anusuya S (2016). Chitosan elicitation for increased curcumin production and stimulation of defence response in turmeric (*Curcuma longa* L.). Ind. Crops Prod..

[18] Saharan, V. *et al*. In International Conference on Advances in Biotechnology (BioTech). *Proceedings*. 23 (2014).

[19] Chandra S (2015). Chitosan nanoparticles: a positive modulator of innate immune responses in plants. Sci. Rep..

[20] Saharan V (2013). Synthesis of chitosan based nanoparticles and their *in vitro* evaluation against phytopathogenic fungi. Int. J. Biol. Macromol..

[21] Saharan V (2016). Cu-Chitosan Nanoparticle Mediated Sustainable Approach To Enhance Seedling Growth in Maize by Mobilizing Reserved Food. J. Agric. Food Chem..

[22] Ahmad, P. *Plant metal interaction: emerging remediation techniques*. (Elsevier, 2016).

[23] Rajasekaran, P. & Santra, S. Hydrothermally treated chitosan hydrogel loaded with copper and zinc particles as a potential micronutrient-based antimicrobial feed additive. *Front. Vet. Sci*. **2** (2015). 10.3389/fvets.2015.00062 PMC4672281 26664989

[24] Brunel F, El Gueddari NE, Moerschbacher BM (2013). Complexation of copper (II) with chitosan nanogels: Toward control of microbial growth. Carbo. Polym..

[25] Bisht S, Balodi R, Ghatak A, Kumar P (2016). Determination of susceptible growth stage and efficacy of fungicidal management of Curvularia leaf spot of maize caused by *Curvularia lunata* (Wakker) Boedijn. Maydica.

[26] Inoue K, Baba Y, Yoshizuka K (1993). Adsorption of metal ions on chitosan and crosslinked copper (II)-complexed chitosan. Bull. Chem. Soc. Japan.

[27] Ngah WW, Endud C, Mayanar R (2002). Removal of copper (II) ions from aqueous solution onto chitosan and cross-linked chitosan beads. Reactive Functional Polym..

[28] Nelson R (1956). A new disease of corn caused by Curvularia maculans. Plant Dis. Reptr.

[29] Akinbode O (2010). Evaluation of antifungal efficacy of some plant extracts on Curvularia lunata, the causal organism of maize leaf spot. African J. Environ. Sci. Technol..

[30] Bowler C, Montagu Mv, Inze D (1992). Superoxide dismutase and stress tolerance. Ann. Rev. Plant Biol..

[31] Gomez‐Vasquez R (2004). Phenylpropanoids, Phenylalanine Ammonia Lyase and Peroxidases in Elicitor‐challenged Cassava (Manihot esculenta) Suspension Cells and Leaves. Ann. Bot..

[32] Fugate KK, Ribeiro WS, Lulai EC, Deckard EL, Finger FL (2016). Cold temperature delays wound healing in postharvest sugarbeet roots. Front. Plant Sci..

[33] Bruce RJ, West CA (1989). Elicitation of lignin biosynthesis and isoperoxidase activity by pectic fragments in suspension cultures of castor bean. Plant Physiol..

[34] Kuźniak E, Urbanek H (2000). The involvement of hydrogen peroxide in plant responses to stresses. Acta Physiol. Plantarum.

[35] Hou J (2013). Identification of quantitative trait loci for resistance to Curvularia leaf spot of maize. Maydica.

[36] Vander P, Vårum KM, Domard A, El Gueddari NE, Moerschbacher BM (1998). Comparison of the ability of partially N-acetylated chitosans and chitooligosaccharides to elicit resistance reactions in wheat leaves. Plant Physiol..

[37] Küpper H (2003). Copper-induced inhibition of photosynthesis: limiting steps of *in vivo* copper chlorophyll formation in Scenedesmus quadricauda. Functional Plant Biol..

[38] Lidon FC, Henriques FS (1991). Limiting step on photosynthesis of rice plants treated with varying copper levels. J. Plant Physiol..

[39] Pätsikkä E, Kairavuo M, Šeršen F, Aro E-M, Tyystjärvi E (2002). Excess copper predisposes photosystem II to photoinhibition *in vivo* by outcompeting iron and causing decrease in leaf chlorophyll. Plant Physiol..

[40] Borkert C, Cox F, Tucker M (1998). Zinc and copper toxicity in peanut, soybean, rice, and corn in soil mixtures. Comm. Soil Sci. Plant Anal..

[41] Kong L, Xie Y, Hu L, Si J, Wang Z (2017). Excessive nitrogen application dampens antioxidant capacity and grain filling in wheat as revealed by metabolic and physiological analyses. Sci. Rep..

[42] Pan S (2013). Roles of plant growth regulators on yield, grain qualities and antioxidant enzyme activities in super hybrid rice (*Oryza sativa* L.). Rice.

[43] Sharvelle, E. G. The nature and uses of modern fungicides. *Nat. Uses Modern Fungicides*. Pp 308 (1961).

[44] Vincent J (1947). Distortion of fungal hyphae in the presence of certain inhibitors. Nature.

[45] Chester KS (1959). How sick is the plant. Plant Pathol..

[46] Wheeler, B. E. J. An introduction to plant diseases. *An Introduction to Plant Diseases*. pp 374 (1969).

[47] Stangarlin J, Pascholati S (2000). Activities of ribulose-1, 5-bisphosphate carboxylase-oxygenase (rubisco), chlorophyllase, β-1, 3 glucanase and chitinase and chlorophyll content in bean cultivars (*Phaseolus vulgaris*) infected with *Uromyces appendiculatus*. Summa Phytopathol..

[48] Adrian WJ (1973). A comparison of a wet pressure digestion method with other commonly used wet and dry-ashing methods. Analyst.

[49] Chance B, Maehly A (1955). Assay of catalases and peroxidases. Methods Enzymol..

[50] Taneja SR, Sachar R (1974). Induction of polyphenol oxidase in germinating wheat seeds. Phytochem..

[51] Moerschbacher BM, Noll UM, Flott BE, Reisener H-J (1988). Lignin biosynthetic enzymes in stem rust infected, resistant and susceptible near-isogenic wheat lines. Physio. Mol. Plant Pathol..

[52] Giannopolitis CN, Ries SK (1977). Superoxide dismutases I. Occurrence in higher plants. Plant Physiol..

[53] IBPGR. Descriptors for maize, International Maize and Wheat Improvement Center, Mexico City/International Board for Plant Genetic Resources, Rome, Pp 100 (1991).

