# Supplementary material for: Cu-chitosan nanoparticle boost defense responses and plant growth in maize (Zea mays L.)
Source: Sci Rep. 2017 Aug 29;7:9754. doi: 10.1038/s41598-017-08571-0 (PMC5575333; doi:10.1038/s41598-017-08571-0)
Supplement: Supplementary file 1 — Supplementary Information [file 41598_2017_8571_MOESM1_ESM.doc]

**Cu-chitosan nanoparticle boost defense responses and plant growth in maize (Zea mays L.)**

Ram Chandra Choudhary**^1^,** R. V. Kumaraswamy**^1^**, Sarita Kumari**^1^**, S. S Sharma^2^, Ajay Pal**^3^**, Ramesh Raliya**^4^,** Pratim Biswas**^4^** and Vinod Saharan***^1^**

^1^ Department of Molecular Biology and Biotechnology, Rajasthan College of Agriculture, Maharana Pratap University of Agriculture and Technology, Udaipur, Rajasthan 313 001, India

^2^ Department of Plant Pathology, Rajasthan College of Agriculture, Maharana Pratap University of Agriculture and Technology, Udaipur, Rajasthan 313 001, India

**^3^** Department of Chemistry and Biochemistry, College of Basic Sciences and Humanities, Chaudhary Charan Singh Haryana Agricultural University, Hisar, Haryana 125 004, India

**^4^** Department of Energy, Environmental and Chemical Engineering, Washington University in St. Louis, MO 63130, USA

**Corresponding author**

E-mail: [vinodsaharan@gmail.com](mailto:vinodsaharan@gmail.com)

Phone: +91-9461180586; Fax: +91-294-2420447

Supplementory information


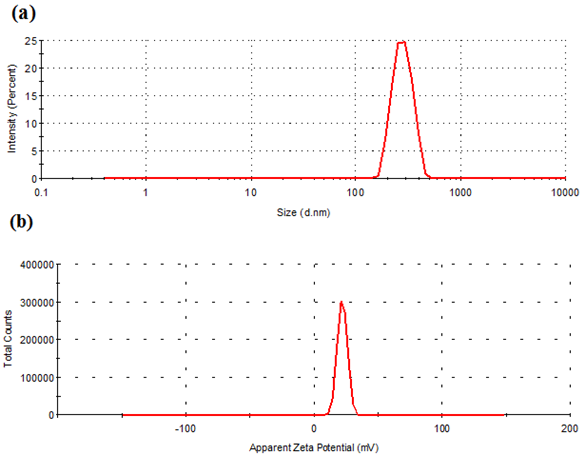


**Figure S1.** DLS analysis of Cu–chitosan nanoparticles (a) size distribution by intensity, and (b) zeta potential distribution.


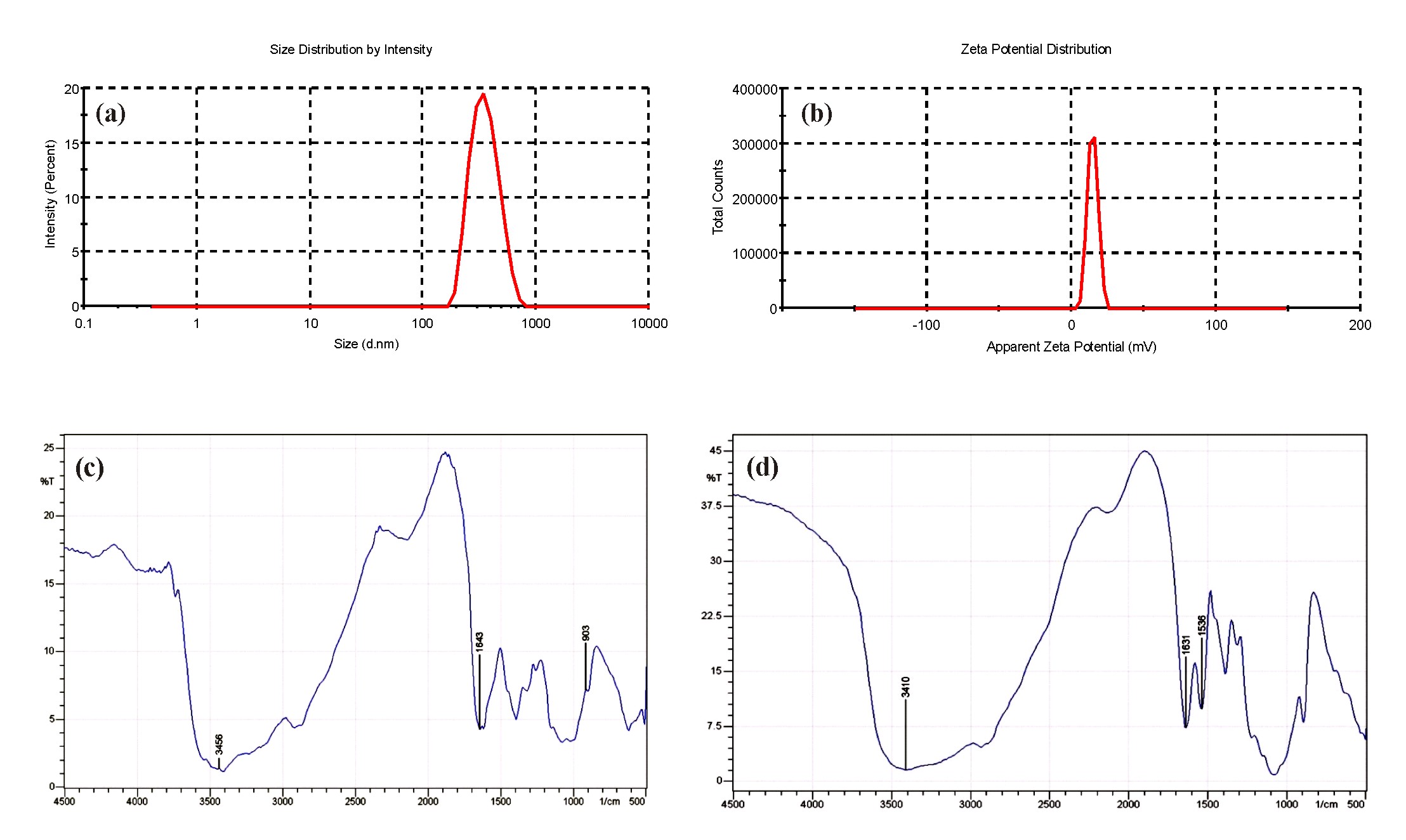


**Figure S2.** DLS analysis of Cu–chitosan nanoparticles (a) size distribution by intensity, and (b) zeta potential distribution. FTIR spectra (c) Bulk chitosan, and (d) Cu–chitosan nanoparticles. X-axis of (c) and (d) is wave number (cm^−1^). *The figure is adopted from reference (Saharan et al 2015)^15^ with copyright permission from Elsevier.*


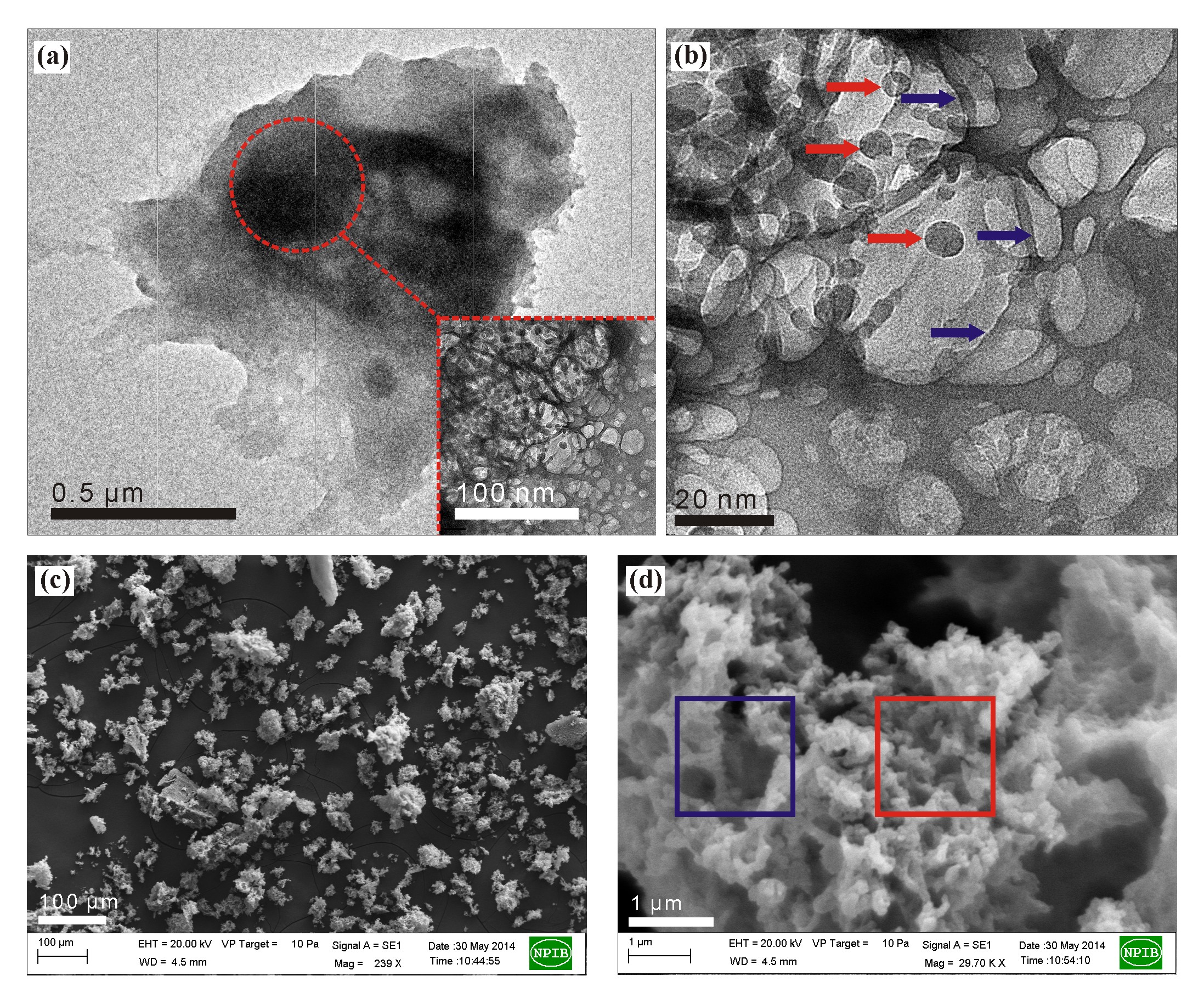


**Figure S3.** TEM micrographs of (a) aggregated Cu–chitosan NPs at 15kx, and at 42kx in inset showing porous network structures. (b) Cu embedded (red arrow) into chitosan porous network (blue arrow) at 110kx magnification. SEM images of (c) Cu–chitosan NPs at 230x, and (d) porous Cu–chitosan at 29.70kx revealed nano (in red rectangular) and micro size pores (in blue rectangular). *The figure is adopted from reference (Saharan et al 2015)^15^ with copyright permission from Elsevier.*


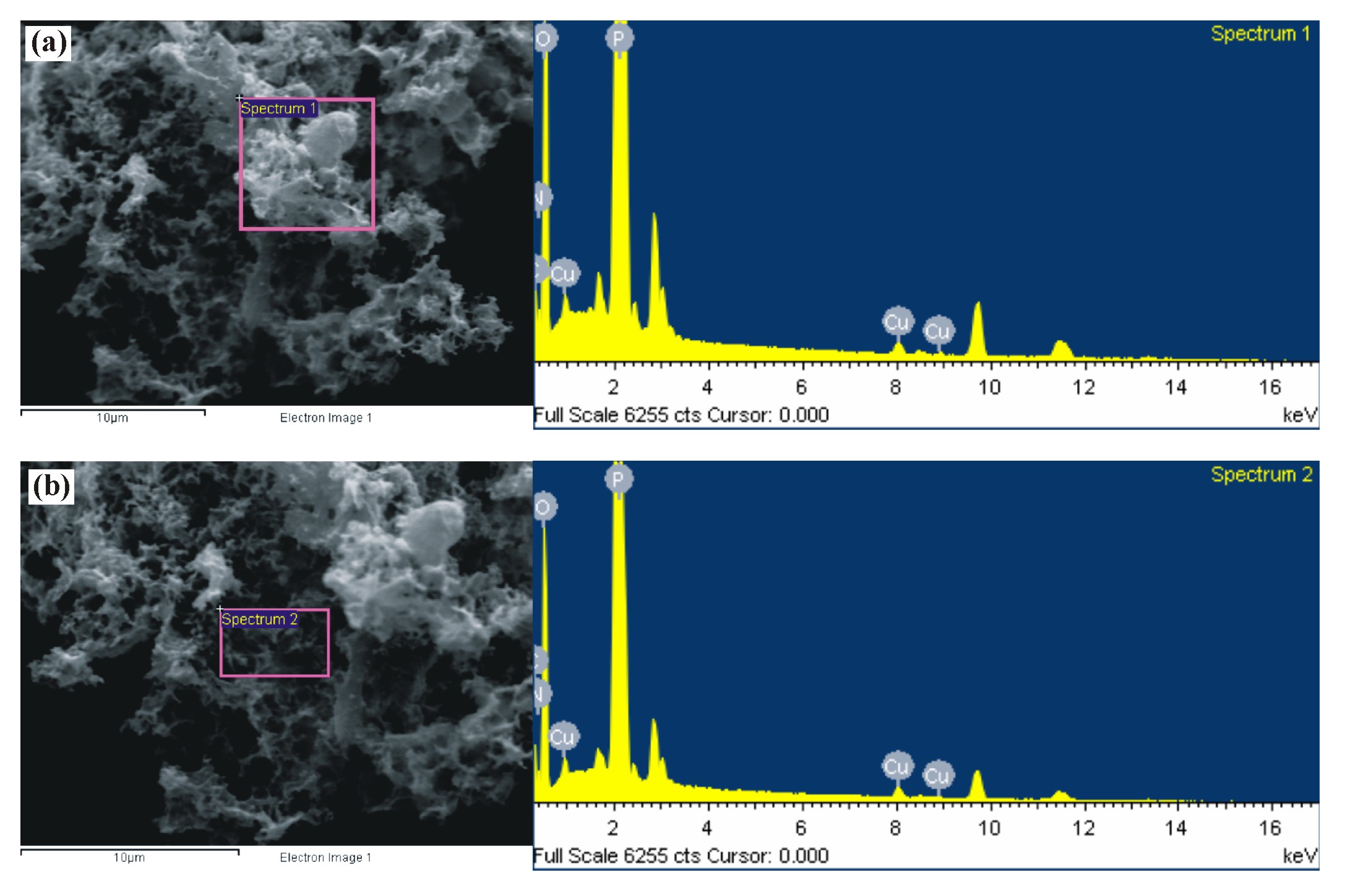


**Figure S4.** SEM-EDX elemental analysis of Cu–chitosan nanoparticles: (a) spectra of non-porous surface, and (b) spectra of porous surface. *The figure is adopted from reference (Saharan et al 2015)^15^ with copyright permission from Elsevier.*


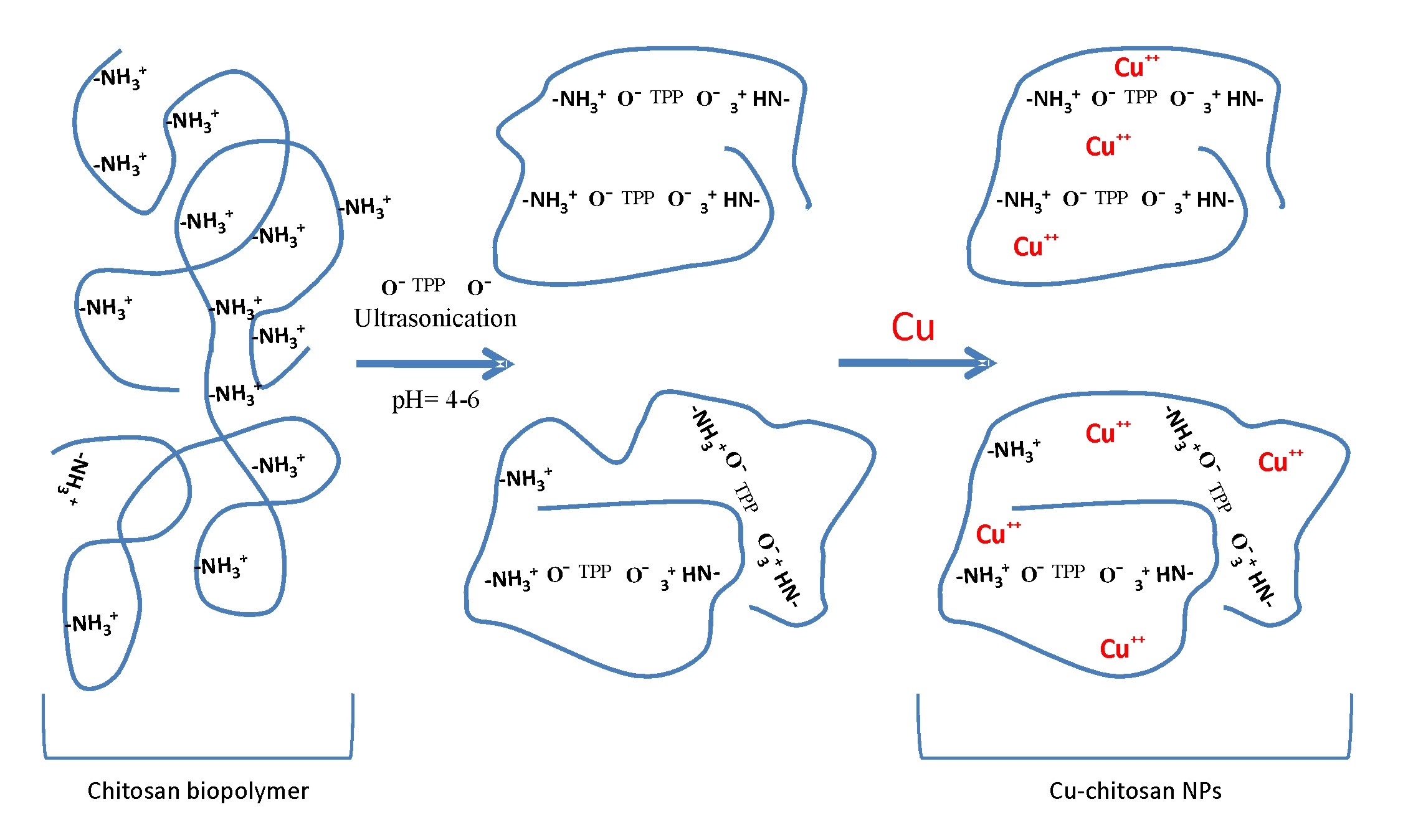


**Figure S5.** Hypothetical model of ionic cross-linking reaction of chitosan, TPP and Cu^2+^ for understanding of structural and synthesis aspects. *The figure is adopted from reference (Saharan et al 2015)^15^ with copyright permission from Elsevier.*

***Table S1.*** *Effect of Cu-chitosan NPs on in vitro mycelial growth of* C. lunata.

| **Treatment (%)** | **% Inhibition of mycelial growth**^A^ |
| --- | --- |
| Control | 00.00±0.00^f^ |
| BCH (0.01) | 21.00±0.80^e^ |
| CuSO_4_ (0.01) | 20.00±0.77^e^ |
| Fungicide (0.01) | 41.00±0.72^b^ |
| Cu-chitosan NPs |  |
| 0.01 | 27.77±0.06^cd^ |
| 0.04 | 28.88±0.35^cd^ |
| 0.08 | 26.66±0.29^d^ |
| 0.12 | 50.00±0.78^a^ |
| 0.16 | 52.70±0.40^a^ |

^A^Each value is mean of triplicates and each replicate consisted of 3 culture plates. Mean ± SE followed by same letter is not significantly different at *p* = 0.05 as determined by Tukey−Kramer HSD. BCH (bulk chitosan, 0.01%) dissolved in 0.1% acetic acid and fungicide (0.01% of Bavistin)


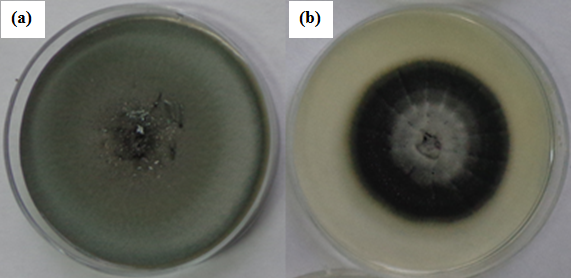


**Figure S6.** Mycelial growth of *C. lunata* in control (a) and in 0.12% Cu-chitosan NPs treatment (b).

**Table S2.** Raw data of Table S1

| **Treatment (%)** | **% Inhibition of mycelial growth** | | |
| --- | --- | --- | --- |
|  | R1 | R2 | R3 |
| Control | 0.00 | 0.00 | 0.00 |
| BCH (0.01) | 19.44 | 21.60 | 22.04 |
| CuSO_4_ (0.01) | 18.51 | 20.40 | 21.09 |
| Fungicide (0.01) | 40.50 | 42.40 | 40.01 |
| Cu-chitosan NPs |  | | |
| 0.01 | 27.69 | 27.73 | 27.90 |
| 0.04 | 28.47 | 28.65 | 29.60 |
| 0.08 | 26.66 | 27.17 | 26.15 |
| 0.12 | 51.55 | 49.40 | 49.05 |
| 0.16 | 53.50 | 52.29 | 52.40 |

**Table S3.** Raw data of Table 2

| **Treatment (%)** | **DS (%)** | | | **PEDC (%)** | | |
| --- | --- | --- | --- | --- | --- | --- |
|  | R1 | R2 | R3 | R1 | R2 | R3 |
| Control (water) | 46.0 | 44.2 | 42.0 | 0.00 | 0.00 | 0.00 |
| BCH (0.01) | 34.0 | 32.0 | 32.1 | 26.09 | 27.27 | 23.81 |
| CuSO_4_ (0.01) | 32.0 | 34.0 | 30.0 | 30.43 | 22.73 | 28.57 |
| Fungicide (0.01) | 30.0 | 30.0 | 28.0 | 34.78 | 31.82 | 33.33 |
| Cu-chitosan NPs |  |  |  |  |  |  |
| 0.01 | 30.0 | 28.5 | 28.5 | 36.36 | 33.33 | 34.78 |
| 0.04 | 24.4 | 25.3 | 24.3 | 47.83 | 40.91 | 42.86 |
| 0.08 | 25.2 | 24.5 | 24.4 | 42.86 | 43.48 | 45.45 |
| 0.12 | 24.0 | 24.0 | 22.0 | 47.83 | 45.45 | 47.67 |
| 0.16 | 23.4 | 22.2 | 21.4 | 47.62 | 50.00 | 47.83 |

**Table S4.** Raw data of Table 3

| **Treatment (%)** | **Cu content (μg/g dw)** | | |
| --- | --- | --- | --- |
|  | R1 | R2 | R3 |
| Control (water) | 4.00 | 4.10 | 3.98 |
| BCH (0.01) | 4.13 | 3.95 | 4.77 |
| CuSO_4_ (0.01) | 25.41 | 23.13 | 23.78 |
| Fungicide (0.01) | 5.12 | 4.07 | 3.92 |
| Cu-chitosan NPs | | | |
| 0.01 | 8.23 | 9.54 | 8.03 |
| 0.04 | 13.06 | 12.10 | 13.15 |
| 0.08 | 16.33 | 15.89 | 16.01 |
| 0.12 | 20.21 | 19.43 | 18.11 |
| 0.16 | 27.91 | 29.56 | 28.20 |

**Table S5.** Raw data of Table 4

| **Treatment (%)** | **DS (%)** | | | **PEDC (%)** | | |
| --- | --- | --- | --- | --- | --- | --- |
|  | R1 | R2 | R3 | R1 | R2 | R3 |
| Control (water) | 64.21 | 62.64 | 65.91 | 0.00 | 0.00 | 0.00 |
| BCH (0.01) | 47.53 | 48.00 | 49.00 | 25.97 | 23.38 | 25.66 |
| CuSO_4_ (0.01) | 46.36 | 48.44 | 47.78 | 27.81 | 22.67 | 27.51 |
| Fungicide (0.01) | 47.11 | 47.33 | 46.53 | 26.63 | 24.44 | 29.40 |
| Cu-chitosan NPs |  |  |  |  |  |  |
| 0.01 | 47.00 | 45.93 | 46.33 | 26.80 | 26.68 | 29.70 |
| 0.04 | 46.00 | 46.57 | 46.22 | 28.36 | 25.67 | 29.87 |
| 0.08 | 46.67 | 45.11 | 45.94 | 27.32 | 27.99 | 30.30 |
| 0.12 | 44.90 | 43.23 | 46.00 | 30.07 | 30.99 | 30.21 |
| 0.16 | 43.77 | 40.33 | 43.37 | 31.84 | 35.62 | 34.20 |

**Table S6.** Raw data of Table 5

| **Treatment (%)** | **Days to 50% tasseling** | | | **Days to 50% silking** | | | **Days to 50% ear leaf senescence** | | | **Number of leaves per plant** | | | **Plant height (cm)** | | | **Ear length (cm)** | | | **Ear weight (kg/plot)** | | | **Grain yield (kg/plot)** | | | **100 grain weight (g)** | | |
| --- | --- | --- | --- | --- | --- | --- | --- | --- | --- | --- | --- | --- | --- | --- | --- | --- | --- | --- | --- | --- | --- | --- | --- | --- | --- | --- | --- |
|  | R1 | R2 | R3 | R1 | R2 | R3 | R1 | R2 | R3 | R1 | R2 | R3 | R1 | R2 | R3 | R1 | R2 | R3 | R1 | R2 | R3 | R1 | R2 | R3 | R1 | R2 | R3 |
| Control (water) | 53 | 54 | 54 | 55 | 56 | 56 | 78 | 80 | 77 | 13.3 | 12.7 | 12.0 | 172.7 | 178.0 | 178.3 | 21.5 | 20.4 | 18.1 | 2.7 | 3.0 | 2.8 | 2.0 | 2.1 | 2.1 | 25.0 | 26.0 | 25.7 |
| BCH (0.01) | 54 | 54 | 55 | 58 | 59 | 59 | 77 | 78 | 78 | 14.0 | 10.7 | 12.0 | 187.0 | 183.7 | 188.0 | 19.3 | 21.4 | 23.3 | 2.7 | 2.6 | 2.8 | 2.6 | 2.1 | 2.1 | 25.1 | 26.1 | 25.9 |
| CuSO_4_ (0.01) | 56 | 53 | 54 | 58 | 58 | 57 | 81 | 81 | 80 | 10.7 | 11.3 | 12.0 | 181.0 | 189.0 | 187.3 | 23.1 | 21.6 | 24.3 | 1.8 | 1.7 | 2.2 | 1.2 | 1.9 | 1.3 | 23.9 | 25.2 | 26.2 |
| Fungicide (0.01) | 55 | 54 | 53 | 56 | 58 | 55 | 80 | 79 | 79 | 11.3 | 11.3 | 11.0 | 195.3 | 185.0 | 189.3 | 19.9 | 24.0 | 23.1 | 2.1 | 2.3 | 1.9 | 1.5 | 1.7 | 1.6 | 27.5 | 26.5 | 26.1 |
| Cu-chitosan NPs | | | | | | | | | | | | | | |  | | | | | | | | | | | | |
| 0.01 | 53 | 53 | 54 | 55 | 58 | 58 | 80 | 82 | 82 | 13.7 | 13.0 | 13.7 | 193.3 | 190.3 | 187.0 | 24.0 | 23.2 | 21.8 | 2.6 | 2.4 | 2.2 | 1.7 | 1.9 | 2.0 | 27.3 | 27.1 | 26.1 |
| 0.04 | 53 | 54 | 53 | 56 | 58 | 56 | 83 | 83 | 80 | 14.3 | 14.0 | 13.0 | 193.7 | 194.3 | 194.1 | 22.0 | 20.0 | 26.0 | 2.6 | 1.9 | 2.6 | 2.0 | 2.2 | 1.2 | 25.2 | 26.4 | 25.7 |
| 0.08 | 55 | 54 | 53 | 58 | 57 | 56 | 84 | 84 | 80 | 17.0 | 15.3 | 15.3 | 195.3 | 203.0 | 196.0 | 26.4 | 28.0 | 25.6 | 2.8 | 2.7 | 2.6 | 2.4 | 2.1 | 2.7 | 25.9 | 27.8 | 27.0 |
| 0.12 | 53 | 55 | 53 | 56 | 55 | 58 | 81 | 81 | 80 | 13.0 | 12.0 | 13.0 | 198.1 | 200.5 | 196.0 | 23.4 | 23.6 | 26.0 | 3.6 | 2.9 | 2.8 | 2.2 | 2.6 | 3.0 | 29.1 | 28.5 | 30.1 |
| 0.16 | 55 | 53 | 53 | 58 | 57 | 56 | 78 | 80 | 81 | 12.7 | 12.0 | 11.3 | 190.7 | 189.3 | 193.0 | 26.1 | 28.0 | 21.9 | 3.0 | 3.1 | 3.2 | 2.5 | 2.6 | 3.0 | 28.9 | 30.9 | 29.6 |

**Table S7.** Raw data of Figure 1

| **pH** | **% Cu release** | | | **Time (h)** | **% Cu release** | | |
| --- | --- | --- | --- | --- | --- | --- | --- |
|  | R1 | R2 | R3 |  | R1 | R2 | R3 |
| 1 | 44.94 | 43.67 | 43.72 | 0 | 10.61 | 9.69 | 10.10 |
| 2 | 32.82 | 30.9 | 31.86 | 24 | 17.81 | 19.31 | 18.42 |
| 3 | 21.67 | 21.43 | 21.49 | 48 | 38.05 | 40.38 | 39.10 |
| 4 | 17.55 | 17.15 | 17.35 | 72 | 57.72 | 61.99 | 58.19 |
| 5 | 13.38 | 13.74 | 13.56 | 96 | 82.61 | 82.48 | 82.52 |
| 6 | 6.23 | 6.11 | 6.17 | 120 | 85.15 | 84.95 | 85.08 |
| 7 | 4.98 | 4.91 | 4.93 | 144 | 85.19 | 84.99 | 84.99 |

**Table S8.** Raw data of Figure 2

| **Treatment (%)** | **SOD**  **(μmol/min/g)** | | | **POD**  **(μmol/min/g)** | | | **PAL**  **(μmol/min/g)** | | | **PPO (μmol/min/g)** | | |
| --- | --- | --- | --- | --- | --- | --- | --- | --- | --- | --- | --- | --- |
|  | R1 | R2 | R3 | R1 | R2 | R3 | R1 | R2 | R3 | R1 | R2 | R3 |
| Control (water) | 0.16 | 0.21 | 0.18 | 14.52 | 10.38 | 12.43 | 0.011 | 0.011 | 0.011 | 0.40 | 0.41 | 0.40 |
| BCH (0.01) | 0.05 | 0.08 | 0.09 | 13.70 | 14.44 | 13.46 | 0.009 | 0.007 | 0.008 | 0.41 | 0.41 | 0.42 |
| CuSO_4_ (0.01) | 0.24 | 0.18 | 0.21 | 14.32 | 14.13 | 15.12 | 0.008 | 0.008 | 0.010 | 0.44 | 0.43 | 0.44 |
| Fungicide (0.01) | 0.18 | 0.16 | 0.15 | 12.97 | 12.49 | 12.66 | 0.009 | 0.008 | 0.008 | 0.37 | 0.38 | 0.40 |
| Cu-chitosan NPs | | | | | | | | | | | | |
| 0.01 | 0.30 | 0.31 | 0.33 | 14.97 | 12.97 | 13.43 | 0.013 | 0.015 | 0.015 | 0.42 | 0.43 | 0.42 |
| 0.04 | 0.32 | 0.34 | 0.33 | 18.52 | 20.08 | 19.20 | 0.015 | 0.013 | 0.014 | 0.46 | 0.45 | 0.46 |
| 0.08 | 0.38 | 0.37 | 0.39 | 19.79 | 19.09 | 19.32 | 0.015 | 0.014 | 0.016 | 0.45 | 0.44 | 0.45 |
| 0.12 | 0.35 | 0.35 | 0.38 | 17.28 | 16.16 | 16.84 | 0.016 | 0.017 | 0.018 | 0.49 | 0.49 | 0.48 |
| 0.16 | 0.42 | 0.41 | 0.39 | 27.04 | 26.00 | 26.46 | 0.022 | 0.021 | 0.022 | 0.49 | 0.50 | 0.49 |

**Table S9.** Raw data of Figure 5

| **Treatment (%)** | **Plant height (cm)** | | | **Stem diameter (cm)** | | | **Root length (cm)** | | | **Root number** | | | **Chlorophyll-a (mg/g)** | | | **Chlorophyll-b (mg/g)** | | |
| --- | --- | --- | --- | --- | --- | --- | --- | --- | --- | --- | --- | --- | --- | --- | --- | --- | --- | --- |
|  | R1 | R2 | R3 | R1 | R2 | R3 | R1 | R2 | R3 | R1 | R2 | R3 | R1 | R2 | R3 | R1 | R2 | R3 |
| Control (water) | 52.2 | 54.3 | 52.2 | 1.1 | 1.4 | 1.0 | 33.4 | 31.1 | 32.5 | 11.9 | 10.0 | 10.1 | 7.6 | 7.5 | 7.5 | 0.36 | 0.36 | 0.37 |
| BCH (0.01) | 54.9 | 55.2 | 54.7 | 1.9 | 1.8 | 2.0 | 29.1 | 32.4 | 31.1 | 12.2 | 12.4 | 10.2 | 8.0 | 8.1 | 8.0 | 0.43 | 0.42 | 0.43 |
| CuSO_4_ (0.01) | 60.1 | 57.8 | 59.9 | 1.2 | 1.4 | 1.6 | 25.4 | 24.3 | 23.1 | 8.0 | 9.0 | 7.9 | 4.6 | 4.5 | 4.5 | 0.20 | 0.21 | 0.20 |
| Fungicide (0.01) | 57.4 | 56.6 | 58.9 | 1.5 | 1.6 | 1.5 | 30.1 | 31.4 | 27.5 | 8.1 | 9.2 | 10.6 | 9.2 | 9.2 | 9.4 | 0.47 | 0.46 | 0.46 |
| Cu-chitosan NPs | | | | | | | | | | | | | | | | | | |
| 0.01 | 59.8 | 61.4 | 62.7 | 2.3 | 1.9 | 2.2 | 34.1 | 36.9 | 35.2 | 14.1 | 15.4 | 12.4 | 15.9 | 15.6 | 17.1 | 1.03 | 1.02 | 1.03 |
| 0.04 | 63.7 | 64.5 | 62.7 | 2.3 | 1.9 | 2.6 | 36.1 | 37.9 | 37.4 | 16.0 | 15.7 | 16.2 | 14.5 | 14.6 | 14.7 | 0.80 | 0.79 | 0.80 |
| 0.08 | 77.7 | 80.1 | 75.5 | 2.4 | 2.3 | 2.4 | 38.3 | 37.1 | 36.9 | 14.5 | 15.2 | 17.1 | 12.6 | 12.6 | 12.6 | 0.71 | 0.71 | 0.71 |
| 0.12 | 71.4 | 69.2 | 70.1 | 2.4 | 2.1 | 1.9 | 35.1 | 36.9 | 33.4 | 14 | 16 | 14.9 | 10.6 | 10.6 | 10.6 | 0.59 | 0.57 | 0.58 |
| 0.16 | 83.1 | 81.9 | 82.4 | 2.4 | 2.5 | 2.0 | 30.1 | 29.5 | 27.9 | 14.0 | 10.8 | 12.1 | 6.8 | 6.8 | 6.8 | 0.35 | 0.34 | 0.35 |
